# Black soldier fly frass as a sustainable organic fertilizer: enhancing productivity of leafy vegetables and soil health in Benin

**DOI:** 10.3389/fpls.2025.1663593

**Published:** 2026-01-19

**Authors:** Carline C. J. Santos, Elie A. Dannon, Honorine H. Bougna Tchoumi, Serge F. Mbokou, Sètondji A. P. Etchiha Afoha, Djana Mignouna, Titilayo D. O. Falade, Olapeju Phorbee, Daniel C. Chougourou, Paul Tematio, Sali A. Ndindeng, Rousseau Djouaka

**Affiliations:** 1 AgroEcoHealth Plateform, International Institute of Tropical Agriculture, Cotonou, Benin; 2 Polytechnic School of Abomey-Calavi, Department of Environmental Management, University of Abomey-Calavi, Abomey-Calavi, Benin; 3 Department of Natural Sciences, National University of Sciences, Technologies, Engineering and Mathematics, Abomey, Benin; 4 Faculty of Sciences and Technologies, University of Abomey-Calavi, Abomey-Calavi, Benin; 5 Department of Agricultural Economics, International Institute of Tropical Agriculture, Accra, Ghana; 6 Department of Foods Safety, International Institute of Tropical Agriculture, Ibadan, Nigeria; 7 Department of Soil Sciences, University of Dschang, Dschang, Cameroon; 8 Department of Quality Control, Africa Rice Center, (AfricaRice), Bouake, Côte d'Ivoire

**Keywords:** black soldier fly, frass, insect, yield, manure, organic

## Abstract

Soil nutrients depletion directly threatens sustainability of food systems by reducing agricultural productivity, degrading ecosystem services, thus increasing the need for external inputs. Frass, a nutrient-rich byproduct of insect farming, is increasingly recognized for its potential in sustainable agriculture. In the current study, we explored the effects of composted Black Soldier Fly (BSF) frass on the growth and yield of *Solanum macrocarpon* (African eggplant) and *Lactuca sativa* (Lettuce), as well as its effect on soil nutrient composition, compared to poultry manure and inorganic fertilizers (NPK and urea). Two field experiments were conducted separately for lettuce and the African eggplants, each involving six treatments and four replicates per crop species. For lettuce, treatments included: BSF frass at 20 t/ha (T1), poultry manure at 20 t/ha (T2), BSF frass at 20 t/ha + 100 kg/ha urea (T3), BSF frass at 10 t/ha + 100 kg/ha urea (T4), NPK (15:15:15) at 200 kg/ha + 100 kg/ha urea as positive control (T01), and unfertilized soil as negative control (T0). For African eggplant, treatments consisted of: BSF frass at 15 t/ha (T1), 10 t/ha (T2), and 5 t/ha (T3), poultry manure at 10 t/ha (T4), NPK (15:15:15) at 200 kg/ha + 100 kg/ha urea as positive control (T01), and unfertilized soil as negative control (T0). Plant growth parameters like plant height, number and size of leaves and yield were analyzed. The results showed that, BSF frass at 20t/ha and 10t/ha significantly performed better than the other fertilizers in promoting plant growth and increasing yield in lettuce (3.53 kg per 3m^2^ and 5.12 kg per 3m^2^ in season 1 and 2, respectively) and of the African eggplant (5.04 kg per m^2^). Yield improvements reached approximately 50% compared with inorganic fertilizer treatments. Post-harvest soil analysis showed significant differences among treatments for African eggplant in nitrogen (N), phosphorus (P), potassium (K), and electrical conductivity (EC), while for lettuce, only soil pH differed significantly. These findings suggest that BSF frass is a viable alternative to both inorganic and organic fertilizers, offering a sustainable solution for improving crop productivity and soil health.

## 1 Introduction

One of the biggest challenges nowadays is sustainable and sufficient food production, both in terms of quality and quantity, to meet growing demands. The increasing demands are driven by both sub-Saharan Africa (SSA) population growth and dietary alterations due to socio-economic factors including urbanization and economic growth (de Bruin et al., 2021, de Vos et al., 2024). However, these increased demands put additional pressure on valuable resources (water, energy, soil, etc.) whilst simultaneously leading to an increase in environmental impact (Eggink et al., 2022) and poor public health outcomes (Lawrence et al., 2019). Concurrently, forty percent of soils in SSA are deficient for nitrogen (N), phosphorus (P), and potassium (K) (Rakotoson et al., 2025; Ludemann et al., 2024). Although the application of mineral fertilizers in few farms, low efficiencies in nutrient use and crop yields have been reported (Liverpool-Tasie et al., 2017; Kihara, 2016). This was attributed to low soil content in organic matter, other micronutrients and high soil acidity (Beesigamukama et al., 2020; Vanlauwe et al., 2015).

In addition, a healthy and sustainable diet should prioritize diverse food groups including vegetables, naturally characterized by high fiber, low fat, sodium, and sugar content (Panwar et al., 2024; Nájera Espinosa et al., 2024). Vegetables significantly contribute to a healthy diet, serving as rich sources of minerals, vitamins, and other essential compounds (Salehi, 2021; Dougnon et al., 2012). In the republic of Benin, the vegetable sector employs thousands of people in urban, peri-urban, and rural areas. Among the thirty vegetables crops produced and consumed in Benin, leafy vegetables such as lettuce (*Lactuca sativa L.)* and African eggplant (*Solanum macrocarpon* L), account for 37% of local production (Houessou et al., 2021).

Lettuce is an important vegetable crop in Africa, particularly in urban and peri-urban areas (Abu et al., 2024). It plays a crucial role in food diversification (Kenny and O’Beirne, 2009) and income generation for small-scale producers. It also offers nutritional and potentially medicinal benefits. In particular, it has anti-inflammatory activity, a cholesterol-lowering effect, and anti-diabetic properties (Kim et al., 2016). These benefits are attributed to the presence of bioactive compounds, such as phenolic acids (caffeic acid and chlorogenic acid), flavonoids (quercetin, kaempferol, anthocyanins, luteolin), fiber, iron, folate, and vitamin C (Kim et al., 2016; Soetan et al., 2010).

The African eggplant, known locally as ‘Gboma’ in the Fon language of Benin, is a traditional leafy vegetable highly valued for its mineral contents and therefore vastly sought after by populations in southern Benin (Adango et al., 2021; Ahouangninou, 2013). The African eggplant contains a substantial amount of nutrients and phytochemical compounds, including saponins, phenols, flavonoids, tannins, vitamins A, B, and C, and proteins, as well as mineral salts like calcium (Ca) and iron (Fe) (Gbaguidi et al., 2015; Ibiam and Nwigwe, 2013; Muhanji et al., 2011). These properties make African eggplant a valuable crop for combating malnutrition among children and pregnant women (Habwe et al., 2008). Beyond its nutritional importance, *S. macrocarpon* also has some medicinal properties (Ossamulu et al., 2014). Its leaves, fruits, and roots have a wide range of medicinal uses, including treating sore throats, acting as a laxative, and addressing cardiovascular diseases, stomach ailments, and other health issues (Nwezoku et al., 2017; Adewale et al., 2015).

Despite the efforts of local producers (rural and urban) in propagating the crop, there is still a supply gap in Benin (Assogba et al., 2022; Houessou et al., 2020). Currently, the crop demand is partly satisfied by the existing vegetables systems, which are facing huge constraints including limited access to quality inputs and equipment (seeds, fertilizer, crop protection products, technologies), lack of secured land and capital, depletion of soil nutrients and beneficial soil organisms, unbalanced pesticides use and limited production knowledge and skills (Houessou et al., 2021).

To address these problems, organic fertilization strategies could be explored. This would contribute to sustainable food production, safety, and security by reducing pesticide exposure, enhancing soil organic matter and, beneficial microbiota, as well as supporting climate change mitigation efforts (Khan et al., 2023; Assogba et al., 2022). Additionally, long-term application of organic amendments improves soil biological functions, physical fertility, and carbon ^©^ sequestration (Aytenew and Bore, 2020; Chen et al., 2018). These practices enhance nutrient cycling, soil stability, and microbial diversity while mitigating heavy metals, and decomposing xenobiotic substances (Khan et al., 2023).

In the last decade, Black soldier fly (BSF) (*Hermetia illucens* L.; Diptera: Stratiomyidae) frass, a byproduct of larval digestion, has showed promising potentials as an organic fertilizer in sustainable agriculture. Being rich in essential nutrients like N, P, and K, frass can enhance soil fertility and crop productivity (Idris et al., 2024; Lomonaco et al., 2024). The composition of frass varies with larval feeding substrates, determining its nutrient content and beneficial microbial populations (Lomonaco et al., 2024). Moreover, BSF technologies can address public health, environmental pollution, and technological problems with their capacity to be used as a sustainable organic waste management solution for cities sanitation. The polyphagous BSF larvae have been demonstrated to feed on a large variety of decomposing organic matter (OM), such as food waste (Surendra et al., 2016), human excreta (Banks et al., 2014; Lalander et al., 2013), fruits waste (Dzepe et al., 2020) and different animal manures, without bearing disease vectors like houseflies (*Musca domestica* L., Diptera: Muscidae).

Previous studies have demonstrated that frass application can improve soil health, promote plant growth, and increase crop yields across various plant species like maize, kales, swiss chard, French beans and tomatoes (Lomonaco et al., 2024; Poveda and González-Andrés, 2021). The use of BSF frass aligns with circular economy principles, offering a sustainable solution for biodegradable waste management while producing valuable agricultural inputs (Lopes et al., 2022).

Also, the combined application of organic and mineral fertilizers has been recommended for improved nutrient use and crop yield (Vanlauwe et al., 2015). However, there is limited knowledge on the effects of BSF frass on leafy vegetable production (including underutilized species like the African eggplant) and widely utilized crop species like lettuce) in comparison with the other organic fertilizers applied in Benin cropping systems. This study was then designed to get insight into the effects of BSF frass on growth, yield in *L. sativa* and *S. macrocarpon* and soils mineral composition after harvest in comparison with other sources of fertilizer.

## 2 Materials and methods

### 2.1 Experimental site

Field experiments were carried out at Republic of Benin cities of Cotonou and Abomey-Calavi (at IITA-Benin station), for two cropping seasons in 2022 and 2024. Cotonou (6°20’N, 2°20’E) is bordered by Nokoué Lake in the north, the Atlantic Ocean in south, the municipality of Sèmè-Kpodji (in the Ouémé department) in east side, and the municipality of Abomey-Calavi in West. Annual cumulated rainfall ranges were 870 mm and 969 mm in 2022, for Cotonou and Abomey-Calavi, respectively (INSAE, 2016). Mean temperature varied between 27 °C and 35 °C and relative humidity between 65% and 85% for both cities (INSAE, 2016). The International Institute of Tropical Agriculture (IITA) is located in Abomey Calavi (6°25’N, 2°19’E). Typically, rainfall during the wet season (from June to October) is 748.9 mm, mean temperatures is 26 to 28 °C and relative humidity is 82% to 85% (Santos et al., 2015). The climate of two cities is subequatorial and receives bimodal rainfall. Soil type is sandy to sandy-loamy characterized by poor organic matter (OM > 2), total nitrogen (N > 0.08%), Phosphorus (P_(ppm)_ > 20), Potassium (K _(meq/100 g soil)_ > 0.4) (Nacoulma and Guigma, 2015; Santos et al., 2015).

### 2.2 Source of fertilizers and preparation

Three fertilizers were used in the experiments: two organic fertilizers (BSF frass and poultry manure) and mineral fertilizer (NPK 15:15:15). The BSF frass was obtained from AgroEcoHealth platform of IITA-Benin (https://www.iita.org/iita-countries/benin), and consisted of residues from fruit waste conversion by BSF larvae. The fruits were ground and sieved for two days in a basket to reduce water content. The resulting mash was then mixed with bovine blood purchased from a slaughterhouse. Eight thousand (8000) five-day old BSF larvae, were then placed in a mixture of 3 kg of fruits waste and blood. Two weeks later, the residues consisting of excrement, exuviae, and transformed substrate were collected, dried for about a month, and used for the experiment. Poultry manure was obtained from poultry raisers and mineral fertilizers (NPK and Urea) purchased from agrochemical shops. Characteristics of the different sources of organic manure included in the present study and unfertilized soil before the experiments for lettuce and the African eggplant were given in **Table 1**.

**Table 1:** Physico-chemical characteristics of soils, BSF frass and chicken manure before application for *Solanum macrocarpon* and *Lactuca sativa* production.

Plant species	Substrate	N (mg/kg)	NO_3_^-^ (mg/kg)	NH_4_^+^ (mg/kg)	Mg (mg/kg)	K (mg/kg)	P (mg/kg)	Ca (mg/kg)	Conductivity (EC)	pH
*Solanum macrocarpon*	Unfertilized soil	0.15	0.64	0.19	12.67	5.1	5.79	29.33	38.14	7.27
	Chicken manure	1.72	7.568	2.236	23.67	8.13	29.83	300	6.31	7.72
	BSF Frass	1.04	4.576	1.352	17	12	29.27	266.67	15.85	6.38
*L. sativa*	Unfertilized soil	0.24	1.056	0.312	0.55	7.85	9	68	40.5	5.30
	Chicken manure	6.7	29.48	8.71	6.3	81	24	290	5.92	7.75
	BSF Frass	5.35	23.54	6.955	9.16	44.1	15	260	7.04	6.35

For *L. sativa*, Chicken manure gave the highest N (6.7 mg/kg), N0_3_^-^ (29.48 mg/kg), NH4^+^ (8.71 mg/kg), K (81 mg/kg), Mg (24 mg/kg), and Ca (290 mg/kg) contents, making it a rich source of essential nutrients. The BSF frass, while lower in nitrogen (5.35 mg/kg), contained the highest phosphorus concentration (9.16 mg/kg), making it particularly beneficial for phosphorus-deficient soils. Unfertilized soil, with significantly lower nutrient levels, shows poor fertility, particularly in nitrogen (0.24 mg/kg) and phosphorus (0.55 mg/kg). The electrical conductivity (EC) was highest in unfertilized soil (40.5), suggesting potential salinity issues, whereas chicken manure (5.92) and BSF frass (7.04) presented more moderate EC values. In terms of pH, chicken manure was slightly alkaline (7.75), while BSF frass was mildly acidic (6.35), and unfertilized soil is the most acidic (5.30). These results indicate that both organic amendments significantly improve soil fertility, with chicken manure providing a more balanced nutrient supply and BSF frass being a better phosphorus source, allowing for targeted soil enrichment based on specific agricultural inputs.

In *S. macrocarpon*, the physico-chemical analysis of the soil and amendments shows significant variations in nutrient composition, which directly influence soil fertility. Chicken manure has the highest nitrogen content (1.72 mg/kg), followed by BSF frass (1.04 mg/kg), while unfertilized soil contains the lowest amount one (0.15 mg/kg). Similarly, nitrate (NO_3_^-^) and ammonium (NH_4_^+^) concentrations were highest in chicken manure (7.568 mg/kg and 2.236 mg/kg, respectively), indicating a greater potential for immediate nitrogen availability. The BSF frass also contained notable amounts of these nitrogen forms (4.576 mg/kg NO_3_^-^ and 1.352 mg/kg NH_4_^+^), whereas unfertilized soil shows minimal levels (0.64 mg/kg NO_3_^-^ and 0.19 mg/kg NH_4_^+^) (**Table 1**). Regarding mineral nutrients, chicken manure and BSF frass had the highest magnesium respectively (23.67 mg/kg; 17 mg/kg), and potassium concentrations (8.13 mg/kg; 12 mg/kg). Both amendments significantly improved phosphorus levels (≈29 mg/kg) compared to unfertilized soil (5.79 mg/kg), which was an essential element for plant growth. In addition, calcium levels were substantially higher in amended soils, with chicken manure (containing 300 mg/kg) and BSF frass (266.67 mg/kg), compared to that (29.33 mg/kg) of unfertilized soil. Electrical conductivity (EC) is highest in BSF frass (15.85), indicating increased dissolved salts, whereas chicken manure exhibits the lowest EC (6.31). The pH values of all treatments remain within a favorable range, with chicken manure slightly alkaline (7.72) and BSF frass slightly acidic (6.38), ensuring nutrient availability (**Table 1**). Overall, both organic amendments significantly enhanced specific soil nutrients content, improving potential soil fertility.

### 2.3 Experimental design and crop management

#### 2.3.1 The lettuce*, Lactuca sativa*

Experiments on *L. sativa* were carried out during the small rainy season in June to August 2022 (season 1) and in June to August 2023 (season 2) at Abomey-Calavi. Lettuce variety sown was “Eden”, widely adopted by producers in Benin for its good agronomic performance and high yield. It was sown in nursery and irrigated for three weeks without fertilizer application. Nursery plot (3m^2^) was irrigated twice per day using 5 L water per m^2^. Fertilizers were applied after transplanting by affecting each type of fertilizer to an experimental plot of 3m^2^. Each experimental plot contained in total 24 transplanted lettuce with 30 cm * 30 cm spacing. Fertilizer treatments consisted of 1) BSF frass at 20 t/ha (T1); 2) poultry manure at 20 t/ha (T2); 3) BSF frass at 20 t/ha + 100 kg/ha Urea (T3); 4) BSF frass at 10 t/ha + 100 kg/ha Urea (T4); 5) inorganic fertilizer containing nitrogen, phosphorus and potassium (NPK15:15:15) 200 kg/ha + urea 100 kg/ha as positive control (T01); and 6) soil with no fertilizer as a negative control (T0). These six treatments were arranged in complete randomized block design with 4 replicates. Fertilizers were applied, one week after transplanting (28 days after sowing). The second fertilizer application was performed two weeks later. Experimental plots were weeded twice during the whole experimental period. Growth parameters measured three time during the plant cycle included plant height and, stem diameter. Four plants were randomly selected in the middle of each experimental plot to estimate growth parameters. At the end of the experiment, plants were harvested per experimental plot to estimate yield. The cumulated rainfall was 559 mm and 802.5 mm in season 1 and season 2, respectively. The mean temperatures were 27.25 ± 0.64 °C and 27.23 ± 1.02 °C in season 1 and season 2, respectively. The relative humidity averaged 81.23 ± 3.42% and 85.44 ± 4.0% in season 1 and season 2, respectively.

#### 2.3.2 The African eggplant, *Solanum macrocarpon*

Experiment using the *S. macrocarpon* was performed in the small rainy season in 2024 at Cotonou. Similarly to lettuce, the African eggplant was sown in nursery on of 3m^2^, watered twice everyday using 2.5 L of water for 3 weeks and the transplanted on 1m^2^ experimental plot. Fertilizer treatments consisted of: 1) BSF frass at 15t/ha (T1); 2) BSF frass at 10 t/ha (T2); 3) BSF frass at 5 t/ha (T3); 4) poultry manure at 10 t/ha (T4); 5) inorganic fertilizer containing nitrogen, phosphorus and potassium (NPK15:15:15) 200 kg/ha + urea 100 kg/ha as positive control (T01); and 6) soil with no fertilizer application as a negative control (T0). Treatments were arranged in complete randomized block design with 4 replicates. Fertilizer application was done for the first time, 1 week after transplanting (28 days after sowing) and the second one, two weeks later. Weeding was performed twice during the whole experiment period. Estimated growth parameters consisted of number of leaves, plant height, leaf length and width. Four plants were randomly chosen to estimate growth parameters during 3 weeks while yield was estimated by harvesting and weighing plants on experimental plots. The African eggplant has been harvested twice (Harvest 1 and Harvest 2) as the plant continued to grow after the first harvest. The cumulated rainfall was 526 mm and the mean temperature was 27.42 ± 1.50 °C. The relative humidity averaged 92.70 ± 2.80% during the experimental period.

### 2.4 Mineral composition of soil samples and organic fertilizers used

The soil and organic fertilizers macronutrients were analyzed at the chemical laboratory of the AgroEcoHealth platform at IITA-Benin. Before planting and after harvesting, soils were sampled at 0–20 cm depth in each experimental plot for soil nutrients analysis, pH and conductivity. To assess the nitrogen (N), phosphorus (P), potassium (K^+^), magnesium (Mg^2+^), calcium (Ca^2+^) content and conductivity of the soil samples, we first extracted them using the protocol described by Mehlich 3 as reported by Wuenscher et al. (2015). It involved weighing 3 g of previously sorted and ground sample, introducing it into a 50 mL tube and adding 30 mL of a solvent solution containing 0.01 M CaCl_2_, 0.25 M NaHCO_3_ and 0.013 M HNO_3_. The mixture was mechanically stirred for 10 min and prior to mineral analysis. 10 mL of the extract was taken and put in a photometer (Wagtech 7100 photometer, UK) at the chemical laboratory of the AgroEcoHealth platform, IITA Benin, to estimate the mineral content and conductivity of the samples.

### 2.5 Data collection

Soil mineral content was determined before planting and after harvesting of each plant species. The growth parameters considered were the number and size of leaves per plant, the height of the plants, the diameter of the clump, and recorded once a week after transplanting on four central plants per experimental plot. At harvest, plants were counted and weighed to estimate yield in kg/m2. A tape measure was used to take height and clump diameter. Yield was estimated just after harvesting.

### 2.6 Statistical analysis

Data on leaf number, plant height, clump diameter, and yield were processed by performing the Analysis of variance (ANOVA) with 95% confidence interval (CI) with the software SAS 9.2 (SAS Institute Inc, 2009). Statistically significant differences between the mean values in different treatments were determined using the Student Newman Keuls method with 95% CI (P<0.05). Principal component analysis (PCA) was performed with R software. Model residuals were verified for normality using Shapiro-Wilk normality test. The test t of Student was applied to compare soil nutrients content before planting and after planting of L. sativa and S. macrocrpon.

## 3 Results

### 3.1 Effect of different fertilizers on the growth of *Lactuca sativa*

Highly significant differences (p < 0.001) were observed between treatments for lettuce growth parameters. **Table 2** gives the effects of different treatments (T0, T01, T1, T2, T3 and T4) on plant height and stem diameter over two seasons at three observation time points (Week 3, Week 4, and Week 5). In Season 1, T2 (poultry manure 20t/ha) and T3 (BSF Frass 20t/ha + 100 kg/ha Urea) gave the highest plant heights at Week 5 (14.93 ± 0.33 cm and 14.31 ± 1.02 cm, respectively), followed by T1 (BSF Frass 20t/ha) and T4 (BSF Frass 10t/ha + 100 kg/ha Urea) (13.87 ± 0.53 cm and 13.45 ± 0.54 cm, respectively), while T01 (200 kg/ha NPK + 100 kg/ha Urea) and T0 (untreated plot) presented the lowest values across all weeks. A similar trend was observed in Season 2, where T3 and T4 showed the greatest heights. For stem diameter, Season 1 recorded higher values, with T1, T2, T3, and T4 significantly outperforming the control (T0: 19.06 ± 0.92 cm *vs*. treated plants >25 cm). However, in Season 2, diameter values were lower, suggesting possible environmental influences. Overall, the results indicated that treatments significantly enhanced plant growth, particularly T2 and T3, while seasonal variations likely affected stem development (**Table 2**).

**Table 2:** Effect of BSF Frass, poultry manure and NPK+Urea on plant height and stem diameter of *L. sativa*.

Traits	Treatments	2022			2023		
		Week 3	Week 4	Week 5	Week 3	Week 4	Week 5
Height (cm)	T0	8.84 ± 0.63^b^	10.48 ± 0.58^b^	11.66 ± 0.49^bc^	8.99 ± 0.48^b^	9.94 ± 0.61^b^	11.63 ± 0.56^ab^
	T01	10.03 ± 0.80^ab^	10.99 ± 0.88^ab^	11.24 ± 0.93^c^	9.79 ± 0.54^ab^	10.53 ± 0.66^ab^	11.01 ± 0.71^b^
	T1	11.66 ± 0.60^ab^	13.36 ± 0.61^ab^	13.87 ± 0.53^ab^	11.19 ± 0.55^a^	12.49 ± 0.64^a^	12.86 ± 0.54^ab^
	T2	11.26 ± 0.73^ab^	12.97 ± 0.65^ab^	14.93 ± 0.33^a^	11.48 ± 0.59^a^	11.41 ± 0.83^ab^	12.41 ± 0.53^ab^
	T3	11.99 ± 1.15^a^	13.91 ± 1.26^a^	14.31 ± 1.02^a^	11.30 ± 0.77^a^	12.59 ± 0.69^a^	13.14 ± 0.83^ab^
	T4	9.75 ± 0.56^ab^	12.61 ± 0.81^ab^	13.45 ± 0.54^abc^	11.39 ± 0.63^a^	12.01 ± 0.66^ab^	13.57 ± 0.54^a^
Stem diameter (cm)	T0			19.06 ± 0.92^b^			9.29 ± 0.48^c^
	T01			20.96 ± 2.17^ab^			9.31 ± 0.36^c^
	T1			26.03 ± 1.36^a^			11.46 ± 0.42^ab^
	T2			26.15 ± 1.10^a^			11.49 ± 0.23^ab^
	T3			26.03 ± 2.12^a^			11.65 ± 0.65^ab^
	T4			25.12 ± 1.11^a^			11.08 ± 0.71^abc^

Same letter in each column indicates no significant differences (P>0.05) between treatments after ANOVA followed by SNK. T1: BSF frass at 20 t/ha; T2: poultry manure at 20 t/ha; T3: BSF frass at 20 t/ha + 100 kg/ha Urea; T4: BSF frass at 10 t/ha + 100 kg/ha Urea; T01: NPK (15:15:15) 200 kg/ha + urea 100 kg/ha; and T0: soil with no fertilizer.

### 3.2 Effect of different fertilizers on the growth of *Solanum macrocarpon*

#### 3.2.1 Plant height

**Figure 1:**
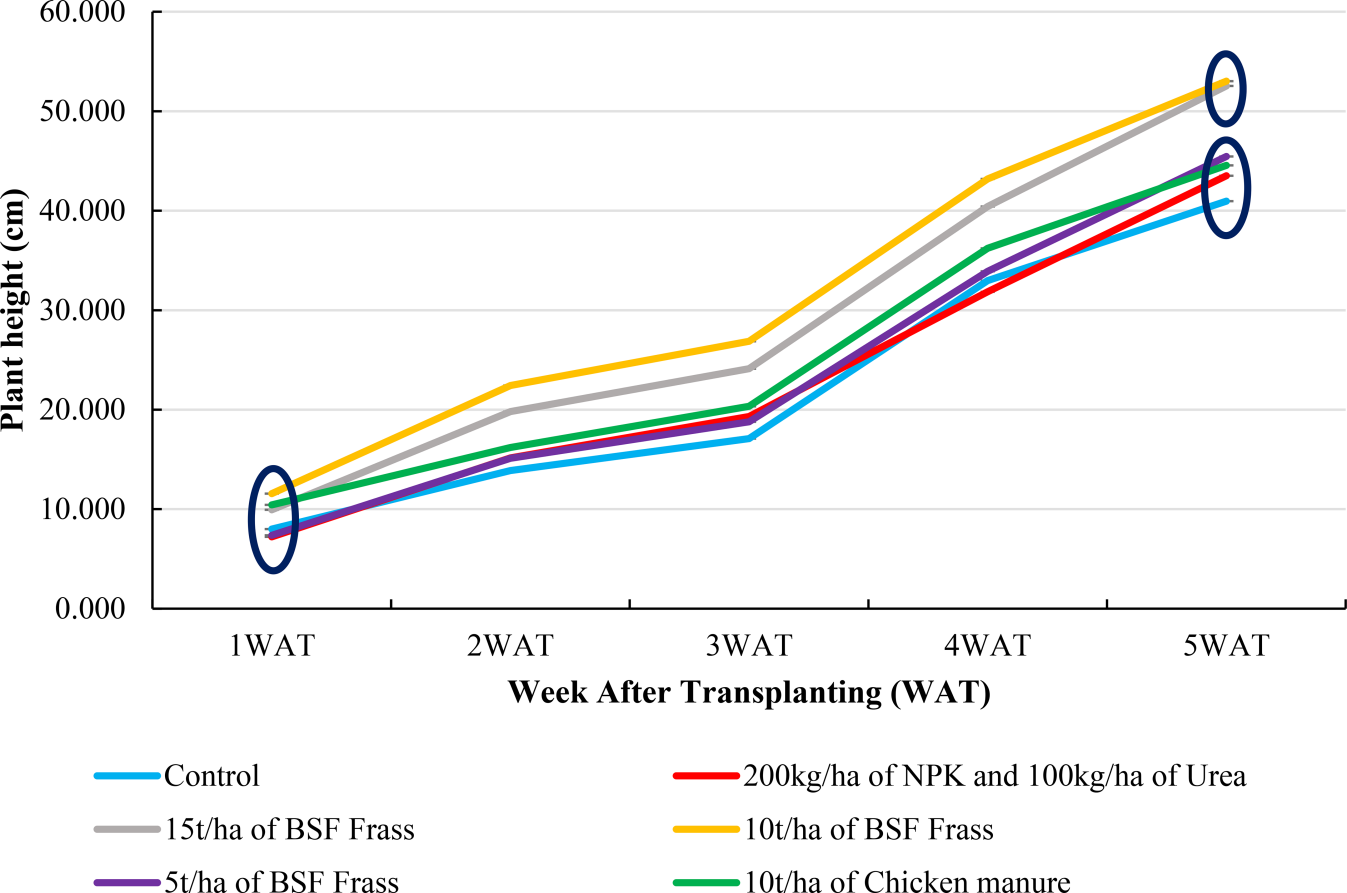
Plant height in *S. macrocarpon* at different sampling dates during harvest 1. WAT: Week After Transplanting.

The curves describing plant height over the development cycle of *S. macrocarpon* revealed that all fertilization treatments have significantly improved plant growth compared to the negative control (water only), with a progressive increase in height over time (**Figure 1**). In particular, curves describing height over time for doses 10 t/ha of BSF frass and 15 t/ha of BSF frass were above those of other treatments, suggesting that these two treatments effectively provided nutrients for *S. macrocarpon* development.

**Figure 2:**
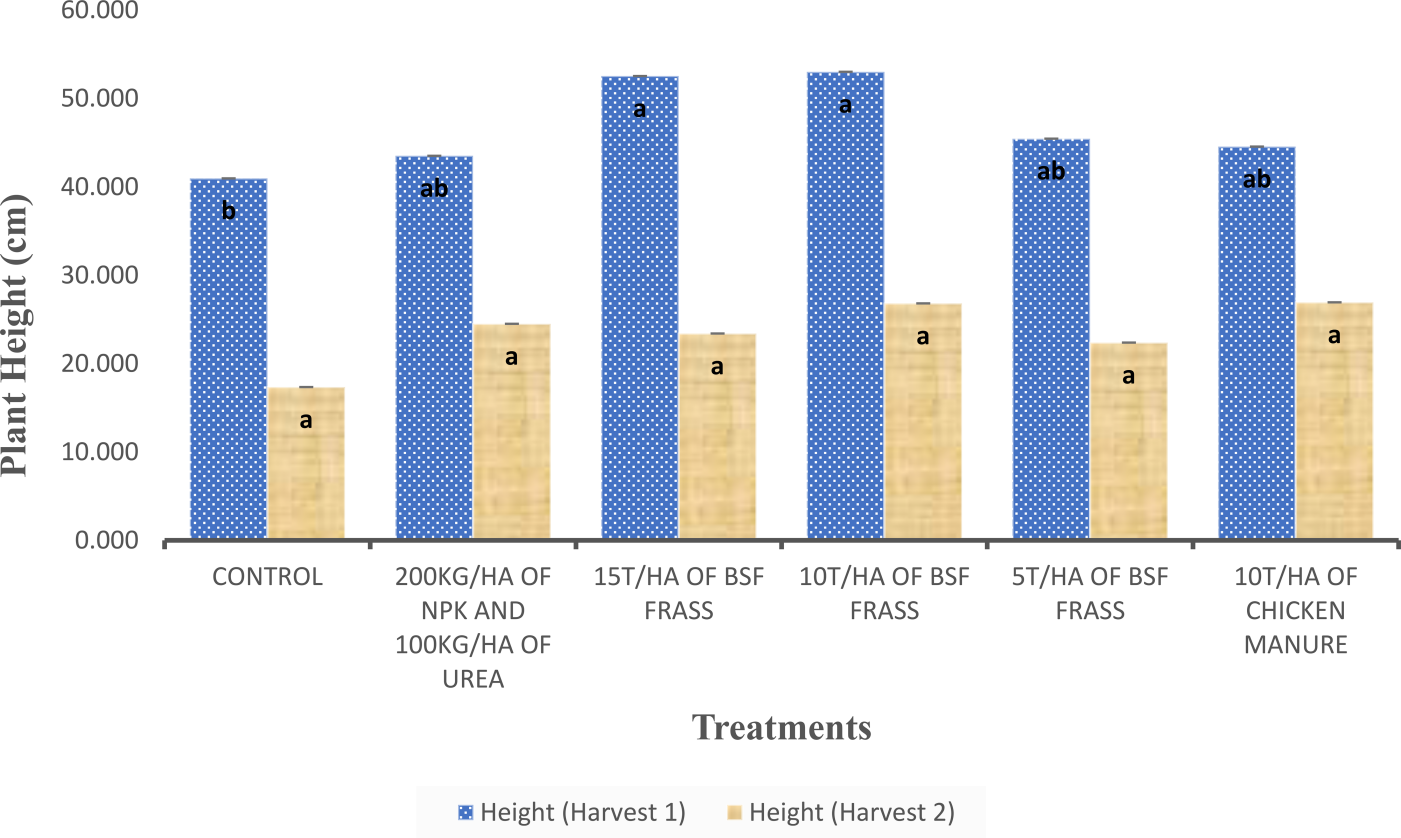
Mean height of *S. macrocarpon* plant measured after application of the different fertilizers. Same letter on top of bars indicate no significant differences (P>0.05) between treatments with ANOVA followed by Student-Newman-Keuls (SNK) test.

The mean plant height was significantly higher for treatments doses 10 t/ha of BSF frass and 15 t/ha of BSF frass compared with the other treatment at Harvest 1 (**Figure 2**). However, no significant differences were observed between the treatments 5t/ha BSF frass, NPK and Chicken manure. The latter treatments did not significantly differ from untreated control. Chemical fertilizers (NPK + Urea) and 5 t/ha of BSF frass showed intermediate growth but have still performed better than water alone. An accelerated growth was observed after July 18 2024, which indicated a key phase of nutrient absorption. Thus, the use of organic amendments, particularly 10 t/ha of BSF Frass, was found to be a promising alternative to chemical fertilizers for optimizing plant growth. At Harvest 2, any significant differences were not observed between treatments (**Figure 2**). Overall, the average plant height was higher in the first harvest than in the second one, regardless of treatments. Treatments including organic and mineral fertilizers generally improved plant growth compared to control. Among the different treatments, the application of 10t/ha of BSF frass resulted in the highest plant height, with 53.01 ± 2.081cm in the first harvest and 26.81 ± 1.36 cm in the second one. The dose 15t/ha of BSF frass treatment follows closely with the previous one, having an average height of 52.53 ± 1.61 cm in the first harvest. On the other hand, the least effective treatment was control, giving the lowest plant height in the first (40.95 ± 1.31 cm) and the second harvest (17.34 ± 1.49 cm). However, all other treatments have shown significant improvement compared to untreated plot. The organic fertilizers including BSF frass and chicken manure were more effective compared to the chemical fertilizer combination (NPK + urea). The use of 10t/ha of BSF frass was found to be the most effective treatment for promoting plant growth.

#### 3.2.2 Number and size of leaves

Leaf production by *S. macrocarpon* followed similar observed for plant height, with T2 (BSF frass at 10t/ha) showing the highest count (45.13 ± 2.29 leaves) in Harvest 1. During Harvest 2, the highest leaf count was observed in T4 poultry manure at 10 t/ha (22.53 ± 1.28 leaves accounting for 50% of that obtained for T2 in Harvest 1), showing that chicken manure also provided a beneficial effect on leaf production over time (**Table 2**). Leaf length varied significantly with treatments, with T2 consistently showing the longest leaves (31.77 ± 0.87 cm in Harvest 1 and 19.63 ± 0.76 cm in Harvest 2). Application of BSF frass at 10 t/ha significantly enhanced leaf expansion, promoting greater biomass accumulation (**Table 3**). Leaf length varied significantly with treatments, with T2 (BSF frass at 10t/ha) consistently showing the longest leaves (31.77 ± 0.87 cm in Harvest 1 and 19.63 ± 0.76 cm in Harvest 2). Application of BSF frass at 10 t/ha significantly enhanced leaf expansion, promoting greater biomass accumulation (**Table 3**). Leaf width followed a similar pattern, where T2 produced the widest leaves (22.42 ± 0.81 cm in Harvest 1 and 11.81 ± 0.53 cm in Harvest 2). Treatments T1 and T4 also performed well, highlighting their effectiveness in promoting leaf development (**Table 3**). 

**Table 3:** Effect of BSF Frass, poultry manure and NPK+Urea on leaf number and size of *Solanum macrocarpon*.

	Treatments	Harvest 1					Harvest 2
		04-July (1 WAT)	11-July (2 WAT)	18-July (3 WAT)	25-July (4 WAT)	01-Aug (5 WAT)	12-Sept (1 MAH1)
Number of leaves	T0	10.03 ± 0.80^a^	14.06 ± 1.06^a^	15.5 ± 1.03^a^	35.69 ± 2.08^a^	37.19 ± 3.41^a^	13.75 ± 1.03^b^
	T01	9.63 ± 0.56^a^	12.75 ± 1.31^a^	16.5 ± 1.60^a^	38.75 ± 1.91^a^	43.94 ± 3.85^a^	14.37 ± 1.13^ab^
	T1	10.25 ± 0.79^a^	13.88 ± 0.94^a^	16.88 ± 1.18^a^	38.38 ± 4.35^a^	46.19 ± 3.11^a^	19.50 ± 1.31^ab^
	T2	11.63 ± 0.47^a^	17.06 ± 0.74^a^	19.56 ± 0.85^a^	39.75 ± 3.01^a^	45.13 ± 2.29^a^	20.87 ± 0.93^ab^
	T3	8.75 ± 0.68^a^	12.94 ± 1.06^a^	16.94 ± 1.48^a^	36.56 ± 3.35^a^	43.88 ± 2.41^a^	18.43 ± 1.07^ab^
	T4	9.27 ± 0.59^a^	12.87 ± 0.85^a^	17.13 ± 0.95^a^	35.93 ± 3.10^a^	46.6 ± 2.90^a^	22.53 ± 1.28^a^
Leaf length (cm)	T0	14.22 ± 0.36^bc^	16.44 ± 0.50^b^	18.91 ± 0.93^b^	23.59 ± 0.92^b^	24.63 ± 1.06^b^	13.16 ± 0.64^b^
	T01	13.11 ± 0.30^c^	16.50 ± 0.55^b^	20.56 ± 0.76^ab^	25.16 ± 0.74^ab^	29 ± 0.98^a^	17.70 ± 0.85^a^
	T1	15.13 ± 0.43^b^	19.25 ± 0.49^a^	22.22 ± 0.68^ab^	26.53 ± 0.77^ab^	30.97 ± 0.83^a^	19.22 ± 0.85^a^
	T2	16.69 ± 0.49^a^	20.50 ± 0.48^a^	29.59 ± 6.04^a^	27.44 ± 0.78^a^	31.77 ± 0.87^a^	19.63 ± 0.76^a^
	T3	13.08 ± 0.45^c^	17.38 ± 0.62^b^	20.66 ± 0.69^ab^	25.50 ± 0.72^ab^	29.59 ± 0.98^a^	18.91 ± 0.64^a^
	T4	13.80 ± 0.50^bc^	17.34 ± 0.57^b^	21.53 ± 0.59^ab^	25.97 ± 0.74^ab^	30.38 ± 0.92^a^	19.66 ± 0.53^a^
Leaf width (cm)	T0	8.83 ± 0.26^bc^	10.55 ± 0.38^ab^	12.63 ± 0.48^a^	17.92 ± 0.47^a^	19.19 ± 0.68^b^	9 ± 0.46^b^
	T01	8.16 ± 0.22^c^	10.19 ± 0.39^b^	13.38 ± 0.52^a^	18.56 ± 0.50^a^	23.66 ± 0.93^a^	12.06 ± 0.58^a^
	T1	9.38 ± 0.27^ab^	12.06 ± 0.35^a^	14.69 ± .52^a^	19.13 ± 0.59^a^	21.69 ± 0.64^a^	12.45 ± 0.49^a^
	T2	9.91 ± 0.31^a^	11.63 ± 0.53^ab^	14.28 ± 0.47^a^	18.81 ± 0.71^a^	22.42 ± 0.81^a^	11.81 ± 0.53^a^
	T3	7.88 ± 0.29^c^	10.59 ± 0.43^ab^	14.34 ± 0.56^a^	18.88 ± 0.60^a^	22.39 ± 0.75^a^	11.95 ± 0.56^a^
	T4	8.17 ± 0.34^c^	10.55 ± 0.47^ab^	14.42 ± 0.66^a^	18.72 ± 0.56^a^	23.06 ± 0.79^a^	10.92 ± 0.51^a^

Same letter in each column indicates no significant differences (P>0.05) between treatments; WAT: week after transplanting, MAH1: month after. harvest 1; T1: BSF frass at 15t/ha; T2: BSF frass at 10 t/ha; T3: BSF frass at 5 t/ha; T4: poultry manure at 10 t/ha; T01: NPK (15:15:15) 200 kg/ha + urea 100 kg/ha; T0: soil with no fertilizer.

### 3.3 Effect of different fertilizers on yield of *Lactuca sativa* (Lettuce) and *Solanum macrocarpon* (African eggplant)

#### 3.3.1 Lactuca sativa

**Figure 3:**
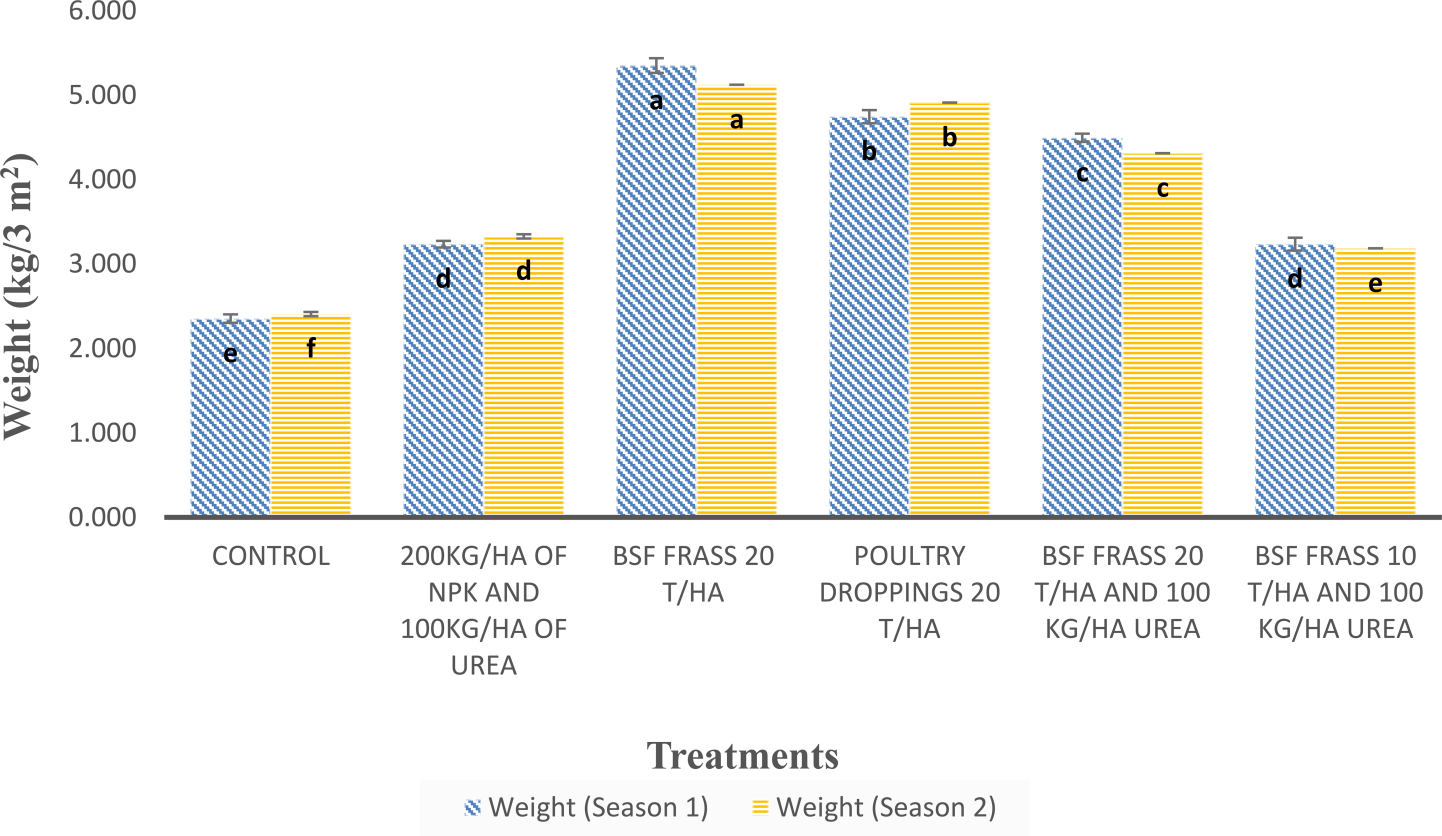
Yield of *L. sativa* under different fertilization treatments across two cropping seasonss. Same letter on top of bars indicate no significant differences (P>0.05) between treatments.

Significantly higher differences (p < 0.001) occurred between treatments for lettuce yield obtained in both season 1 (2022) and season 2 (2023) (**Figure 3**). The BSF frass at 20 t/ha (T1) produced the highest yield (5.35 and 5.12 kg/3m² respectively), followed by poultry manure at 20 t/ha (T2) (4.75 and 4.91 kg/3m²), compared to mineral fertilizers (T01: NPK + Urea) and the untreated control (T0). The lower performance of T3 and T4 (BSF frass + Urea) compared to BSF frass alone suggests potential nutrient imbalance. Yearly or seasonal variations were minimal, indicating the stability of organic amendments in sustaining soil fertility.

#### 3.3.2 Solanum macrocarpon

**Figure 4:**
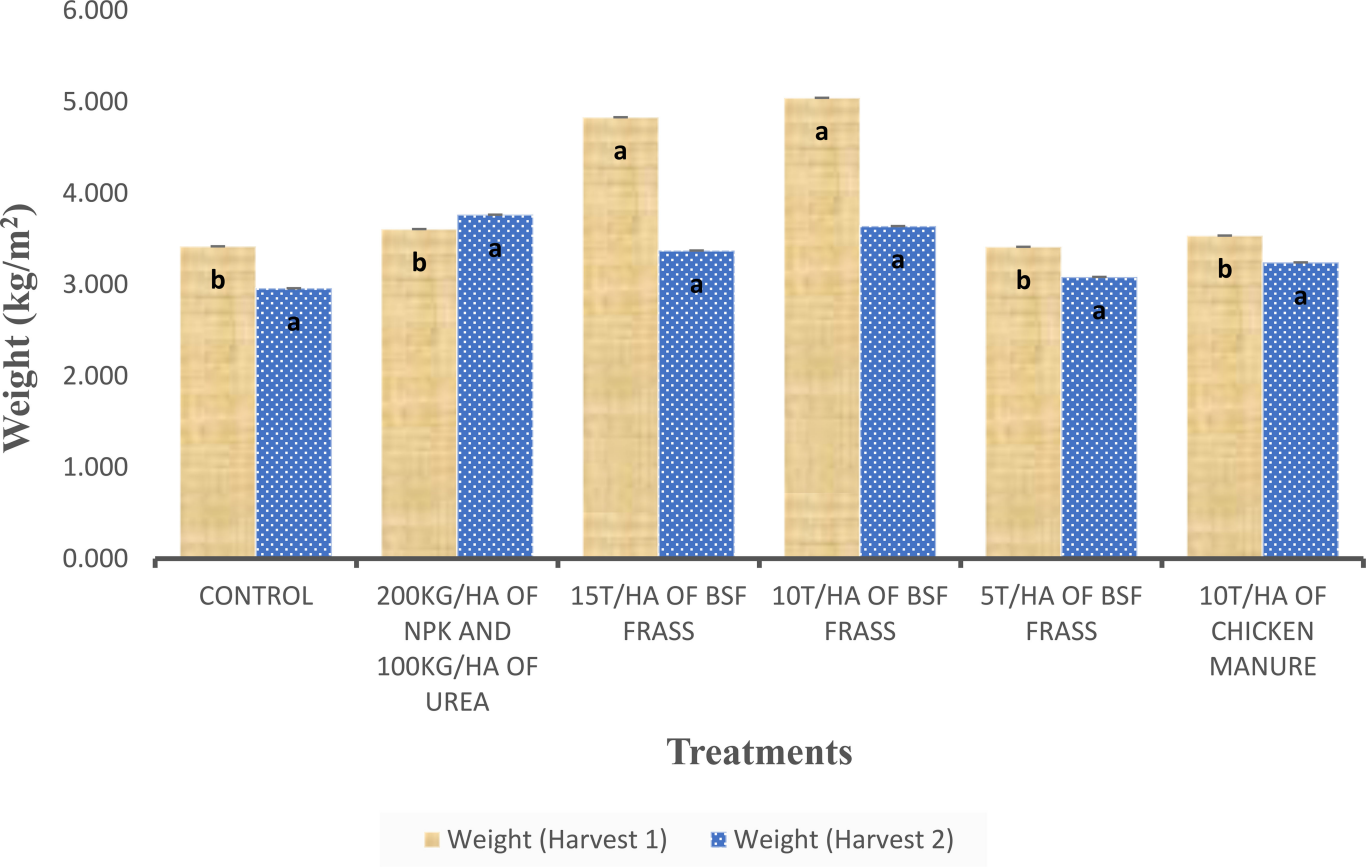
Yield of *S. macrocarpon* yield under different fertilization treatments across two harvests. Same letter on top of bars indicate no significant differences (P>0.05) between treatments.

Significantly higher differences in yield of *S. macrocarpon* grown under different fertilizer treatments (p < 0.001) were observed during the first harvest (**Figure 4**). During this harvest, amendments with BSF frass at 10 t/ha and 15 t/ha produced the highest African eggplant yields (5.04 kg/m² and 4.83 kg/m², respectively) compared to the other treatments. However, treatment with BSF frass at 5 t/ha resulted in a yield similar to those obtained with chicken manure (10 t/ha) and NPK + urea, ranging from 3.41 to 3.60 kg/m. On the other hand, in the second harvest, no significant differences (p>0.05) in African eggplant yield were observed (**Figure 4**). Unfertilized plots produced the lowest yields, while the treatment with 200 kg/ha of NPK and 100 kg/ha of urea showed relatively stable performance across both harvests. In conclusion, BSF frass—especially at 10 to 15 t/ha—proved highly effective in enhancing African eggplant yield, performing better than conventional organic and inorganic fertilizers.

### 3.4 Soil physico-chemical characteristics after transplanting of *Lactuca sativa* and *Solanum macrocarpon*

#### 3.4.1 Lactuca sativa

##### 3.4.1.1 Nitrogen content (N, NO_3_^-^, NH_4_^+^) after harvesting of lettuce

**Figure 5:**
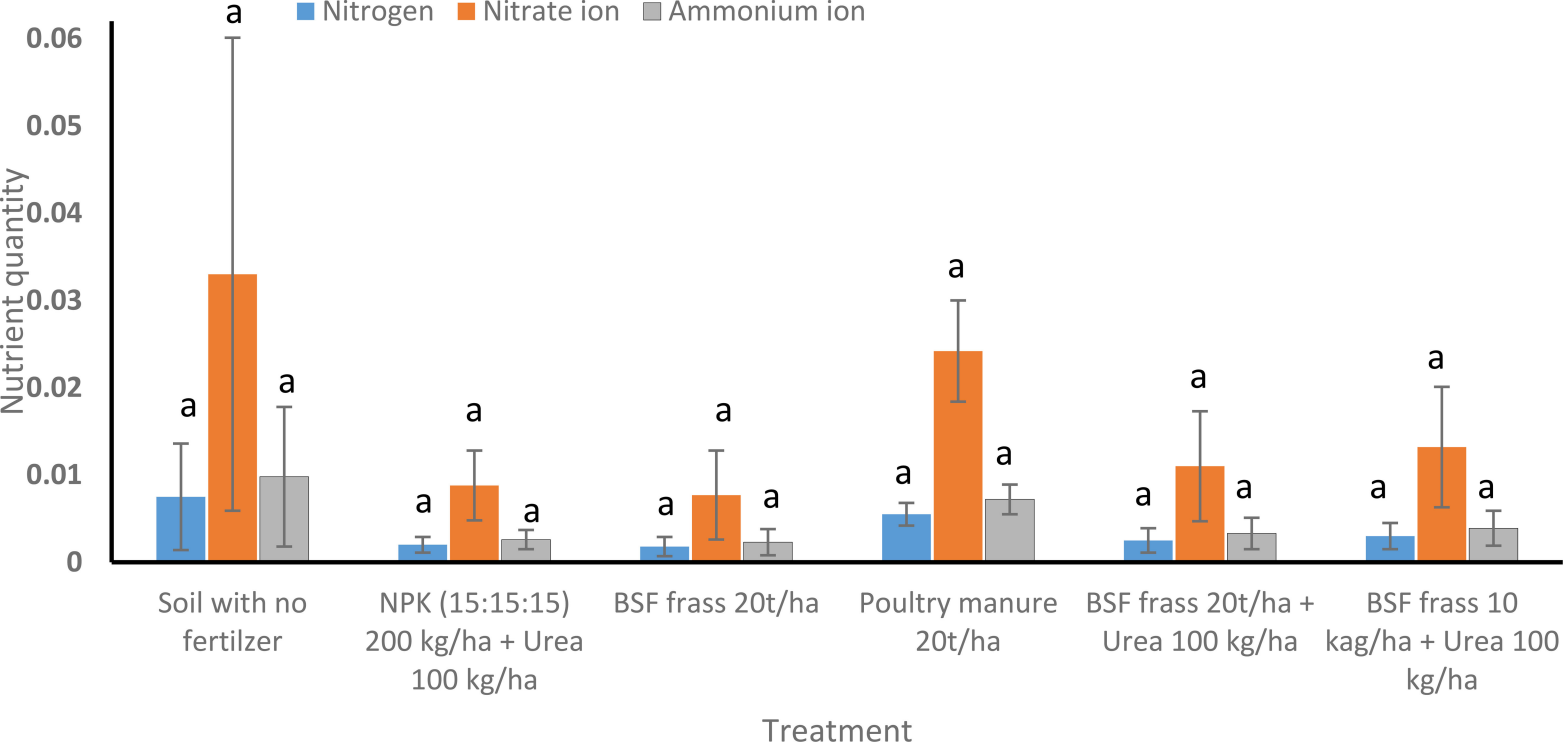
Effect of BSF Frass, poultry manure and NPK + Urea on soil nitrogen concentrations after *Lactuca sativa* harvesting. Bars followed by the same letter indicate no significant differences (P>0.05) between treatments after ANOVA followed by SNK. Error bars are standard errors of the means.

No significant differences were observed between the different treatments after harvesting of *L. sativa*, regardless of nitrogen form (N, N0_3_^-^ and NH4^+^) (**Figure 5**). Poultry manure 20 t/ha (T2) gave higher N0_3_^-^ (0.0242 ± 0.0058 mg/kg) levels, suggesting enhanced nutrient release from organic matter during the mineralization process.

##### 3.4.1.2 Other characteristics of soil after lettuce harvesting

Macronutrients concentrations, electrical conductivity (EC), and pH did not vary significantly between treatments, excepted for P (**Table 4**). However, Poultry manure 20 t/ha (T2) gave higher P (0.4350 ± 0.0384 mg/kg). The inorganic fertilizer application (T01: 200 kg/ha of NPK and 100 kg/ha of urea) presented lower P availability (0.2425 ± 0.0209 mg/kg), possibly due to fixation. The BSF frass combined with urea (T3: 20 t/ha + 100 kg/ha urea) significantly increased K (30.800 ± 14.592 mg/kg).The BSF frass combined with urea (T3: 20 t/ha + 100 kg/ha urea) increased Mg (34.75 ± 17.475 mg/kg), and Ca (42 ± 18.583 mg/kg) contents, indicating its potential to improve soil nutrient balance. But this treatment also exhibits the high EC (126.4 ± 41.268 S/m). Despite these variations, soil pH remained relatively stable (4.82–5.06), suggesting that none of the treatments cause significant acidification or alkalization. Overall, the results highlighted the potential benefits of organic amendments, particularly poultry droppings and BSF frass, in enhancing soil fertility while maintaining nutrient equilibrium.

**Table 4:** Effect of BSF Frass, poultry manure and NPK + Urea on other soil characteristics after *Lactuca sativa* harvesting.

Treatments	P (mg/kg)	K (mg/kg)	Mg (mg/kg)	Ca (mg/kg)	pH	Conductivity (EC)
T0	0.3350 ± 0.0712^ab^	8.450 ± 1.540^a^	12.5 ± 3.708^a^	29 ± 29^a^	4.91 ± 0.1181^a^	45.125 ± 5.864^a^
T01	0.2425 ± 0.0209^b^	5.450 ± 0.236^a^	22 ± 3.464^a^	59.5 ± 20.006^a^	4.825 ± 0.0523^a^	55.325 ± 5.433^a^
T1	0.3325 ± 0.0217^ab^	6.350 ± 0.705^a^	17.5 ± 4.907^a^	27.5 ± 13.022^a^	5.035 ± 0.2522^a^	79.875 ± 6.175^a^
T2	0.4350 ± 0.0384^a^	5.525 ± 1.037^a^	24 ± 5.066^a^	49 ± 21.748^a^	4.9975 ± 0.1293^a^	86.025 ± 11.216^a^
T3	0.3500 ± 0.0408^ab^	30.800 ± 14.592^a^	34.75 ± 17.475^a^	42 ± 18.583^a^	5.0625 ± 0.0949^a^	126.4 ± 41.268^a^
T4	0.3050 ± 0.0266^ab^	11.150 ± 4.424^a^	18 ± 5.671^a^	68 ± 2.309^a^	4.99 ± 0.1142^a^	82.050 ± 16.478^a^

Same letter in the table indicate no significant differences (P>0.05) between treatments after ANOVA followed by SNK. T1: BSF frass at 20 t/ha; T2: poultry manure at 20 t/ha; T3: BSF frass at 20 t/ha + 100 kg/ha Urea; T4: BSF frass at 10 t/ha + 100 kg/ha Urea; T01: NPK (15:15:15) 200 kg/ha + urea 100 kg/ha; and T0: soil with no fertilizer.

#### 3.4.2 Solanum macrocarpon

##### 3.4.2.1 Soil nutrients concentrations after harvesting

###### 3.4.2.1.1 Nitrogen content (N, NO_3_^-^, NH_4_^+^) after *S. macrocarpon* harvesting

**Figure 6:**
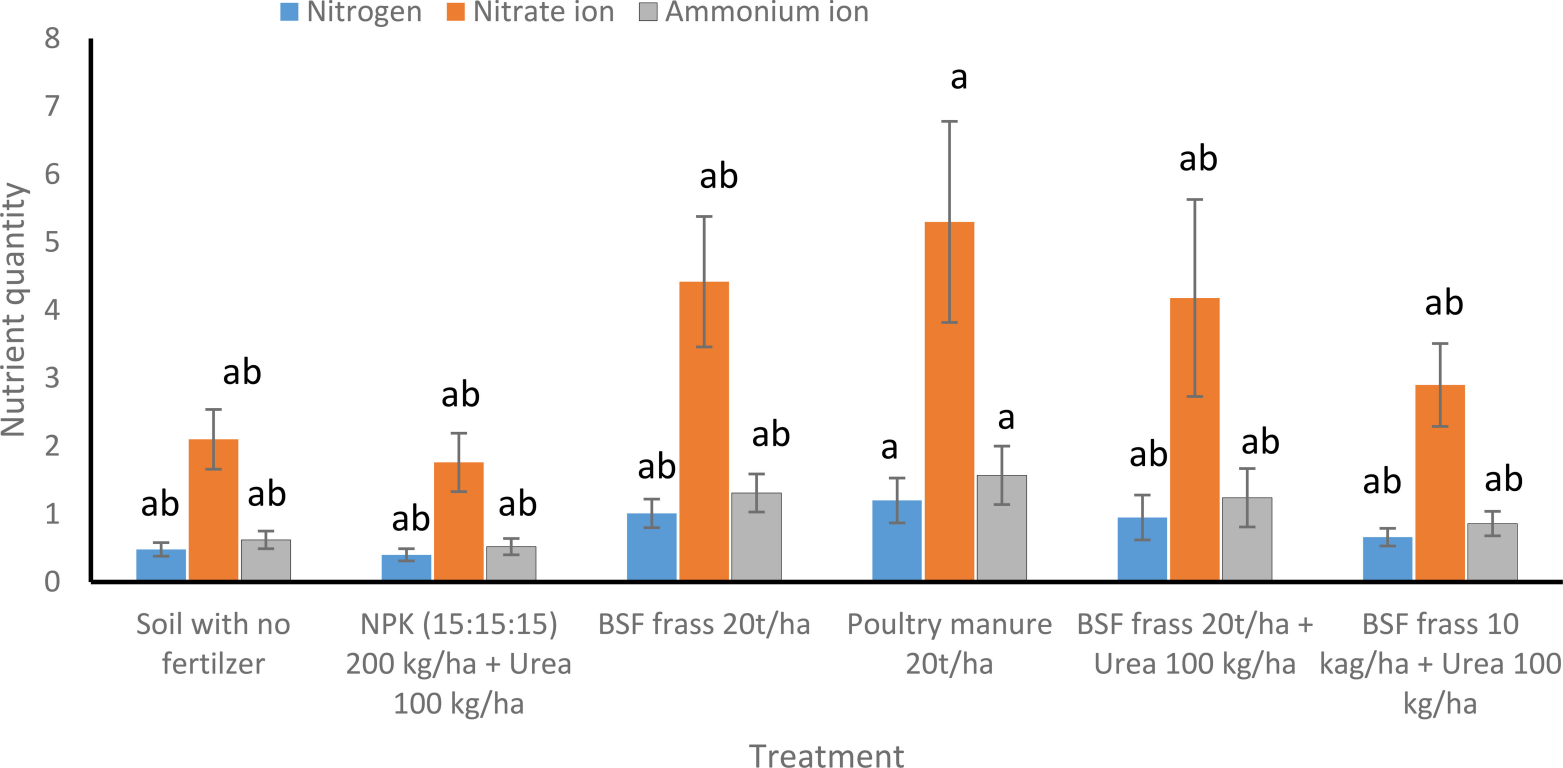
Effect of BSF Frass, poultry manure and NPK + Urea on soil nitrogen concentrations after *Solanum macrocarpon* harvesting. Bars followed by the same letter indicate no significant differences (P>0.05) between treatments after ANOVA followed by SNK. Error bars are standard errors of the means.

There was slight increase in the different forms of nitrogen in Chicken manure, but not significantly different from the other organic fertilizers (**Figure 6**). The nitrogen is a key nutrient for plant growth, available in different forms such as total N, NO_3_^-^, and NH_4_^+^. Higher total N content was observed in treatment T2 (1.20 ± 0.33 mg/kg), followed by T1 (1.01 ± 0.21 mg/kg) and T3 (0.95 ± 0.33 mg/kg), indicating that BSF frass amendments significantly contributed to soil nitrogen enrichment. The NO_3_^-^ levels were higher in T2 (5.30 ± 1.48 mg/kg), suggesting increased microbial nitrification activity, while NH_4_^+^ content follows similar trend (1.57 ± 0.43 mg/kg in T2). Lower N levels are recorded in T01 (0.40 ± 0.09 mg/kg), suggesting that synthetic fertilizers provided a short-term nitrogen boost but may not enhance organic nitrogen accumulation.

###### 3.4.2.1.2 Other characteristics of soil after *S. macrocarpon* harvesting

The magnesium (Mg) content varied slightly among treatments with values ranging from 2.50 ± 1.07 mg/kg (T4) to 4.75 ± 2.95 mg/kg (T1), but was not significantly affected by organic or inorganic amendments (**Table 6**). Similar trend was observed for, P, K, Ca, and EC. Only the pH was significantly more neutral in T1 (BSF frass at 15t/ha and T2 (BSF frass at 10t/ha) while not significantly different compared with that of Chicken manure.

### 3.5 Multivariate analysis of vegetable growth, yield, and soil nutrients

#### 3.5.1 Lactuca sativa

**Figure 7:**
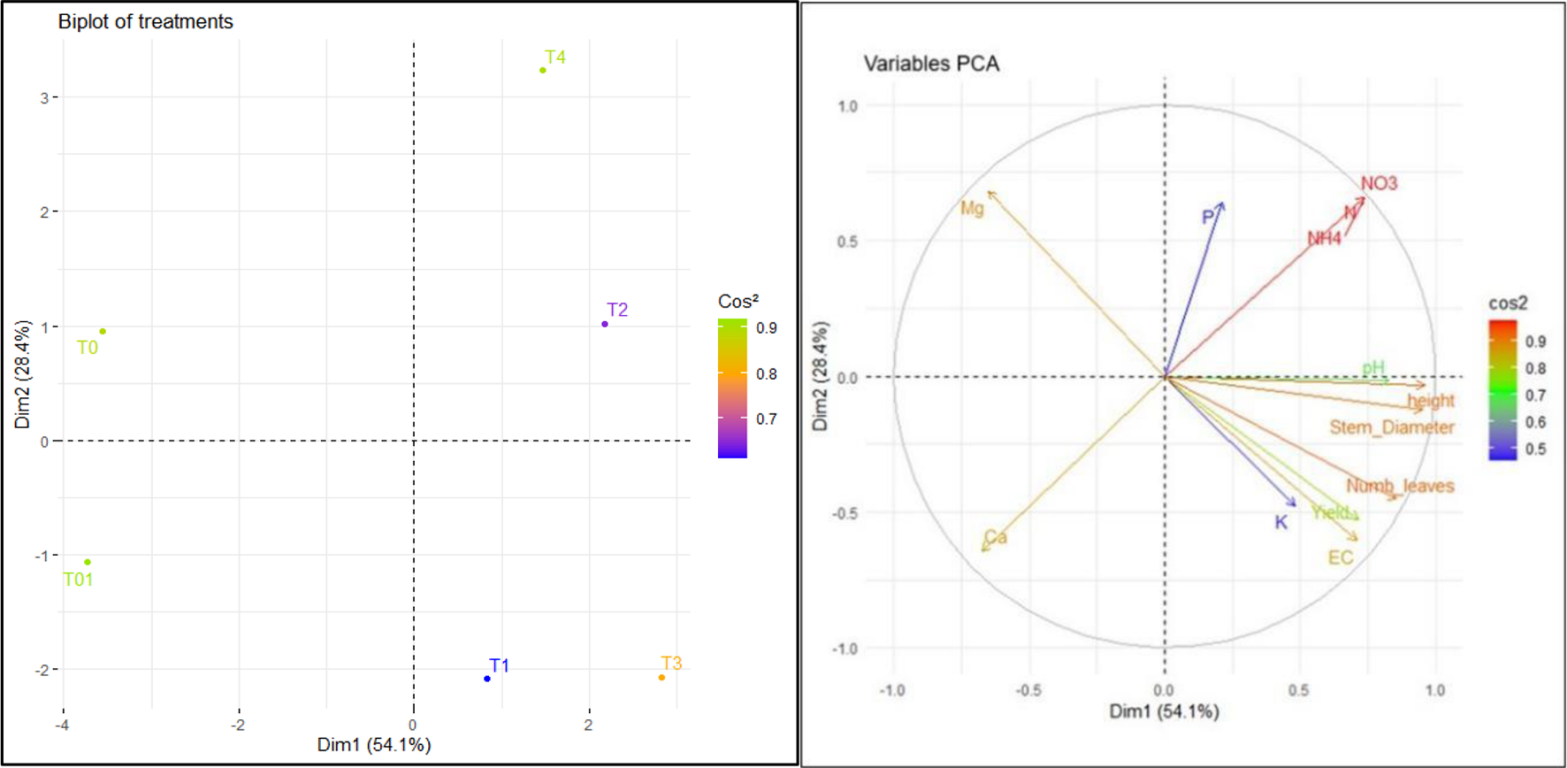
Relationship between different treatments and measured variables based on Principal Component Analysis (PCA): Projection of treatments and variables in the factorial axis system for *Lactuca sativa.* T1: BSF frass at 20 t/ha; T2: poultry manure at 20 t/ha; T3: BSF frass at 20 t/ha + 100 kg/ha Urea; T4: BSF frass at 10 t/ha + 100 kg/ha Urea; T01: NPK (15:15:15) 200 kg/ha + urea 100 kg/ha; and T0: soil with no fertilizer. Ca: calcium, K: potassium, P: phosphorus, Mg: magnesium, EC: Electrical conductivity, pH: hydrogen potential, N: nitrogen, NO_3_: nitrate, NH_4_: ammonium, Dim: dimensions.

Principal Component Analysis (PCA) performed on the agro-morphological parameters of *L. sativa* and the physico-chemical properties of the soil shows that the first two principal components explain 82.5% of the total variance, ensuring a good representation of the data (**Figure 7**). Dimension 1, which explains 54.1% of the total variance, primarily captures variables related to plant nutrition and growth. The variables best explained by this dimension NO_3_^-^, NH_4_^+^, EC, yield, number of leaves, stem diameter, and height. These variables are positively correlated with each other and contribute significantly to Dim 1, suggesting that this component represents a gradient of overall plant vigor or nutrient availability, particularly related to nitrogen and growth performance. The dimension 2 (Dim2) differentiates the treatments based on their P and Ca content. Moreover, Dim2 discriminated treatments according to the balance between Ca and Mg content. Conversely, soils with low nitrogen content, such as treatments T0 and T01 (control without fertilization and mineral fertilizers), negatively affected plant growth and productivity. The BSF frass treatments (T1, T3 and T4) and chicken manure (T2) significantly contributed to plant growth and nutrient availability.

#### 3.5.2 Solanum macrocarpon

**Figure 8:**
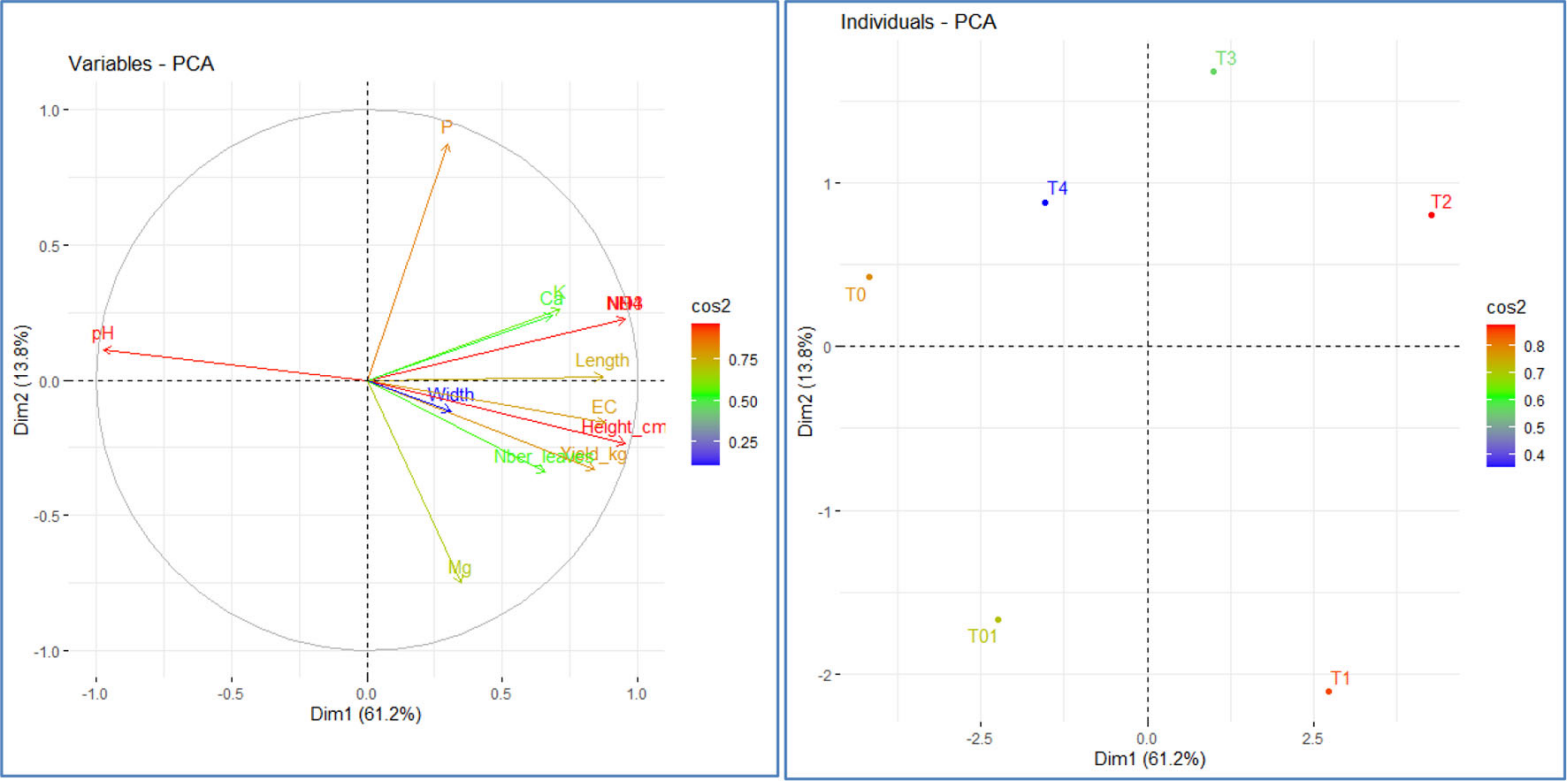
Relationship between different treatments and measured variables based on Principal Component Analysis (PCA): Projection of treatments and variables in the factorial axis system for *Solanum macrocarpon.* T1: BSF frass at 15t/ha; T2: BSF frass at 10 t/ha; T3: BSF frass at 5 t/ha; T4: poultry manure at 10 t/ha; T01: NPK (15:15:15) 200 kg/ha + urea 100 kg/ha; T0: soil with no fertilizer. Ca: calcium, K: potassium, P: phosphorus, Mg: magnesium, EC: Electrical conductivity, pH: hydrogen potential, N: nitrogen, NO_3_: nitrate, NH_4_: ammonium, Dim: dimensions.

Principal Component Analysis (PCA) applied including the following variables the plant growth and yield parameters of *S. macrocarpon* and selected physico-chemical soil parameters revealed that the first two axes (Dim 1 and Dim2) explained 74.99% of the total variance (**Figure 8**). Dimension 1 separates the treatments along a fertility and yield gradient. Treatments with high levels of N, NO_3_^-^, NH_4_^+^, and EC, such as T1 (BSF Fras 15t/ha) and, even more so, T2 (BSF Fras 10t/ha), led to better performance in terms of yield and plant growth. On the other hand, soils with a higher pH, such as the untreated control T0, appear to limit yield, suggesting that slightly acidic soils favor plant growth. Dim2 differentiates the treatments based on the balance between P and Mg. The BSF Frass treatment of 5t/ha was rich in P, while T1 displayed higher Magnesium content, pointing at an inverse relationship: an increase in soil Mg content corresponds to a decrease in available P (**Figure 8**).

### 3.6 Comparison of soils characteristic before planting and after harvesting of *L. sativa* and *S. macrocarpon* using the test t of Student

#### 3.6.1 L. sativa

Comparison of soils N; NO3, NH4 and P contents revealed significantly higher content in all treatments after harvesting of lettuce. The Mg content was higher in treatments T0, T01; T1 T2 and T4 after harvesting while K and Ca contents were higher only in T01. Soils conductivity was higher after harvesting in T1, T2, T4 with a trend to higher value in T01. Soils pH were lower in T0, and T01 with a trend to low value in T2 and T4 after harvesting (**Table 5**).

**Table 5:** Comparison of soils characteristic before planting and after harvesting of *S. macrocarpon* and *L. sativa* using the test t of Student.

Crops	Treatments	t-value and p	N	NO3	NH4	Mg	K	P	Ca	EC	pH
African eggplant	T0	t	2.55	2.55	2.55	1.92	0.18	1.17	0.78	3.53	1.65
		p	0.0513	0.0513	0.0513	0.11	0.87	0.29	0.51	0.036	0.196
	T01	t	1.99	1.99	1.99	1.63	1.26	1.80	0.43	4.39	0.03
		p	0.1	0.1	0.1	0.16	0.26	0.13	0.68	0.02	0.97
	T1	t	3.26	3.26	3.26	1.39	1.7	0.17	0.39	6.86	2.07
		p	0.02	0.02	0.02	0.22	0.18	0.877	0.72	0.006	0.12
	T2	t	3.09	3.09	3.09	1.67	0.85	2.19	0.9	7.87	1.86
		p	0.049	0.049	0.049	0.16	0.45	0.08	0.41	0.004	0.12
	T3	t	2.39	2.39	2.39	1.92	0.93	3.67	0.49	3.11	1.16
		p	0.09	0.09	0.09	0.11	0.42	0.01	0.67	0.05	0.33
	T4	t	2.98	2.98	2.98	1.86	1.57	2.12	0.37	3.46	0.52
		p	0.03	0.03	0.03	0.19	0.18	0.09	0.73	0.04	0.64
Lettuce	T0	t	29.72	37.66	38.40	3.22	0.39	10.57	1.34	0.79	7.72
		p	<0.0001	<0.0001	<0.0001	0.048	0.72	0.002	0.27	0.49	0.03
	T01	t	28.97	255.49	214.79	6.19	10.15	10.72	0.42	2.73	4.90
		p	<0.0001	<0.0001	<0.0001	0.008	0.002	0.002	0.69	0.07	0.003
	T1	t	28.88	199.22	178.06	3.45	2.13	10.61	3.10	6.8	1
		p	<0.0001	<0.0001	<0.0001	0.04	0.12	0.002	0.053	0.008	0.36
	T2	t	28.35	175.55	160.13	4.63	2.24	10.48	0.87	4.06	1.98
		p	<0.0001	<0.0001	<0.0001	0.02	0.11	0.002	0.45	0.03	0.09
	T3	t	28.64	163.20	150.88	1.96	1.57	10.58	1.40	2.08	1.90
		p	<0.0001	<0.0001	<0.0001	0.14	0.21	0.002	0.30	0.13	0.11
	T4	t	28.50	148.87	139.30	3.08	0.75	10.64	0.0	2.52	2.21
		p	<0.0001	<0.0001	<0.0001	0.054	0.51	<0.0001	1.0	0.045	0.07

For Lettuce: T1: BSF frass at 20 t/ha; T2: poultry manure at 20 t/ha; T3: BSF frass at 20 t/ha + 100 kg/ha Urea; T4: BSF frass at 10 t/ha + 100 kg/ha Urea; T01: NPK (15:15:15) 200 kg/ha + urea 100 kg/ha; and T0: soil with no fertilizer. For African Eggplant: T1: BSF frass at 15t/ha; T2: BSF frass at 10 t/ha; T3: BSF frass at 5 t/ha; T4: poultry manure at 10 t/ha; T01: NPK (15:15:15) 200 kg/ha + urea 100 kg/ha; T0: soil with no fertilizer. P: Probabilty: t: test t of student.

**Table 6:** Effect of BSF Frass, poultry manure and NPK + Urea on other soil characteristics after *Solanum macrocarpon* harvesting.

Treatments	P (mg/kg)	K (mg/kg)	Mg (mg/kg)	Ca (mg/kg)	Conductivity (EC)	pH
T0	3.67 ± 1.13^a^	5.21 ± 0.59^a^	7.31 ± 1.03^a^	21.83 ± 1.83^a^	84.43 ± 12.98^ab^	7.34 ± 0.03^a^
T01	4.75 ± 1.53^a^	4.48 ± 0.40^a^	7.08 ± 0.49^a^	25.50 ± 3.57^a^	120.43 ± 18.64^a^	7.28 ± 0.04^ab^
T1	4.75 ± 2.95^a^	6.23 ± 0.65^a^	5.94 ± 0.60^a^	24.17 ± 9.14^a^	148.48 ± 15.97^a^	7.17 ± 0.04^c^
T2	4.42 ± 1.73^a^	6.09 ± 1.15^a^	9.50 ± 1.38^a^	41.67 ± 9.50^a^	148.98 ± 13.96^a^	7.17 ± .0.04^c^
T3	3.25 ± 1.65^a^	6.74 ± 1.76^a^	9.24 ± 0.71^a^	24.67 ± 1.98^a^	145.79 ± 34.54^a^	7.21 ± 0.05^ab^
T4	2.50 ± 1.07^a^	4.64 ± 0.23^a^	8.27 ± 0.92^a^	26.00 ± 3.86^a^	89.42 ± 14.70^ab^	7.29 ± 0.03^ab^

Same letter in the table indicate no significant differences (P>0.05) between treatments after ANOVA followed by SNK. T1: BSF frass at 15t/ha; T2: BSF frass at 10 t/ha; T3: BSF frass at 5 t/ha; T4: poultry manure at 10 t/ha; T01: NPK (15:15:15) 200 kg/ha + urea 100 kg/ha; T0: soil with no fertilizer.

#### 3.6.2 S. macrocarpon

Comparison of the mineral content of soils before and after amendment with the different organic fertilizers included in this study revealed significant differences in all treatments for the African eggplant except for Mg, K, Ca and pH values. In the control treatment T0, soils contents in N, NO3, NH4 and conductivity were higher after harvesting compared to the characteristics of soil before planting. In T01, only conductivity showed significant differences with higher value after harvesting. Soils content in N, NO_3_-, NH_4_+ and conductivity were significantly higher after harvesting. Similar findings were observed in T2, and T4. In addition, the P content was significantly higher in these treatments after harvesting. In T3, there was a trend of higher values of N, NO_3_- and NH_4_+ after harvesting and for P content in T2 and T4 (**Table 5**).

## 4 Discussion

Farming systems in countries of SSA rely mostly on inherent soil fertility with very little inputs of mineral fertilizers because of their high costs and unavailability from a local source (FAO, 2017). Likewise, the use of organic manures is still limited largely because of their low availability due to other competing uses on the farm, such as feeding of animals (Rusinamhodzi et al., 2016) and domestic use as fuel (Ndambi et al., 2019). Such competing uses leave little or none of the organic resources for use in crop production. Therefore, improving soil productivity using organic resources requires a venture into new organic fertilizer sources (Beesigamukama et al., 2020). In recent years, one of the promising solutions to enhance the availability of organic fertilizers in sub-Saharan Africa is the use of insect by-products such as the composted black soldier fly residues called “frass” used in this study (Klammsteiner et al., 2020).

### 4.1 Lactuca sativa

For lettuce production, the results indicated that organic and combined organic-inorganic amendments significantly enhanced the growth performance compared to control treatments. Specifically, poultry manure (T2) and BSF frass combined with urea (T3) led to the highest plant heights and stem diameters in both seasons, with notable seasonal variations. These findings align with previous studies highlighting the beneficial effects of organic amendments on crop growth. For instance, Adekiya et al. (2019) reported that poultry manure significantly improved lettuce height and biomass accumulation due to its high nutrient content and slow-release properties. Similarly, Liu et al. (2024) demonstrated that organic fertilizers enhance soil structure and microbial activity, thereby improving nutrient uptake efficiency in leafy vegetables.

A key observation in our study is the effect of seasonal variations on plant growth, particularly for stem diameter, which was lower in Season 2. This is consistent with findings by Sun et al. (2022), who noted that environmental factors such as temperature and precipitation affect plant morphological traits. The lower stem diameter in Season 2 observed, even with the soil amendments, may be attributed to fluctuations in temperature or moisture availability, impacting nutrient uptake and allocation.

The graph (**Figure 7**) comparing total yield per 3m² across different treatments over two seasons revealed highly significant differences (p < 0.001). BSF frass applied at 20 t/ha (T1) produced the highest yield (5.35 and 5.12 kg/3m²), followed by poultry droppings at 20 t/ha (T2) (4.75 and 4.91 kg/3m²). These results demonstrated that at sufficient doses organics amendments could be used singly (without inorganic fertilizers) for lettuce production. This result confirmed those from Sawinska et al. (2024) and Houben et al. (2020) who supported that frass can be as effective in fertilization as classic NPK mineral fertilizers because of its quick mineralization and easily available nutrients and can supplement or even completely replace mineral fertilization.

The higher performance of BSF frass (T1) aligns with findings by Chavez et al. (2023) on lettuce, arugula and tomatoes. Van Huis et al. (2021) reported that insect frass improves nutrient availability and microbial activity, thereby promoting plant productivity. According to Watson et al. (2021) and Klammsteiner et al. (2020), treating frass may also be good to increase nitrogen availability, through anaerobic digestion or composting. Frass can also have a stimulating effect on the number of soil microorganisms: the number of bacteria, archaea and fungi, carbon mineralization and nitrification, which may ultimately translate into the availability of post-food nutrients for the plant. The lower performance of T3 (BSF frass at 20 t/ha + 100 kg/ha Urea) and T4 (BSF frass at 10 t/ha + 100 kg/ha Urea) compared to BSF frass alone suggests potential nutrient imbalances, as previously noted by Elissen et al. (2023), who highlighted the importance of balancing N sources in organic-inorganic fertilization systems. In addition, BSF frass doses (10 t/ha and 20t/ha) already contained enough mineral N to meet the nutrient needs of the lettuce plant. Therefore, the addition of Urea created a N surplus that disrupted the chemical properties of the soil resulting in inhibition of plant growth and reduction of plant yields. This could be due to the toxicity of NH₄⁺. This effect may also be allelopathic (Gärttling et al., 2020). According to Alromian et al. (2020), frass had various effects on the yield and mineral composition of lettuce leaves. Also, no increase in the yield was observed even at higher doses.

In terms of post-harvest soil nutrient concentrations, P levels were significantly higher in poultry manure-treated soils (T2) followed by T1 (BSF frass), while inorganic fertilizer (T01) gave the lowest P availability. This result supports the work of Mensah and Frimpong (2018), who reported that organic amendments, particularly poultry manure, enhance P solubility through microbial mineralization, whereas synthetic fertilizers can lead to phosphorus fixation, reducing plant availability. Likewise, Chavez et al. (2024) reported lower soluble P concentrations in the frass treatments.

Similarly, BSF frass combined with urea (T3: BSF frass 20t/ha+ 100 kg/ha of urea) significantly increased K, Mg, and Ca concentrations, suggesting its potential to improve soil nutrients balance. These results agree with studies by Van Huis et al. (2021), who found that insect frass contributes to soil fertility by enhancing cation exchange capacity and providing essential macronutrients. Likewise, Fernández-Romero et al. (2016) observed the impacts of BSFL frass on lettuce production and found increases in soil organic matter and residual nutrient content. In the case of fertilizing the Gongronema latifolium Benth (Apocynaceae) plant with inorganic NPK fertilizer, this led to an increase in the concentration of P, sodium (Na), Ca, Mg, P and N in the leaves; the concentration of nutrients increases with increasing fertilization (Osuagwu et al., 2010). However, electrical conductivity (EC) was highest in T3, indicating potential risks of salinity buildup leading decrease lettuce yield observed in this treatment. Watson et al. (2022) mentioned nitrite (N02) build-up, emissions of substantial quantities of CO2 and N2O, salinity and ammonia content of the frass. Consequently, decreases in inorganic fertilizer application can reduce subsequent environmental problems, such as soil degradation and eutrophication.

Soil pH remained stable but acidic across treatments probably due to overuse of chemical fertilizers promote soil acidification by releasing H⁺; and intensive farming and monoculture. The soil of IITA-Benin had been used in rice production with a higher application of chemical fertilizers leading to reduced soil fertility and crop yields and increased toxicity of metals such as aluminum (Al) and manganese (Mn), which are harmful to crops (Mensah and Frimpong, 2018).

Moreover, Mg accumulation depends on many factors. These include genetic factors, but also environmental factors such as soil and climate. The plant absorbs nutrients from the soil solution through various mechanisms. Potassium, calcium and magnesium ions have antagonistic effects. The uptake of P by a plant depends on its concentration in the soil solution, and the concentration also affects the uptake mechanism. The role of P in the plant growth was multiple and it mainly, as a support for photosynthesis. It also increases the plant's resistance to unfavorable environmental conditions (water shortage) by regulating the opening of stomata. Calcium in the plant is responsible for the construction of cell walls. The results of PCA supported these conclusions on effects of cations exchanges on lettuce growth and yield.

In the conducted experiment, frass application had influenced the fresh mass of lettuce and did not confirm those from Sawinska et al. (2024). More research is needed on different frass types (and related to the larval feedstock and possible thermal pre-treatment) and in long-term field experiments with a variety of crops with special emphasis on C and N mineralization. Lopes et al. (2022) have compiled data on 17 BSF frass samples and concluded that frass composition is highly variable and dependent on substrate, especially P, K and micronutrient concentrations. Palma et al. (2020) also concluded that the frass needs stabilization before application as soil amendment. Seasonal variations in plant growth were observed, particularly for stem diameter, but total yield remained stable across seasons, reinforcing the long-term viability of organic amendments as part of sustainable food systems (FAO, 2017).

### 4.2 Solanum macrocarpon

In African eggplant, our results demonstrated that fertilizer treatments significantly affected the growth parameters and yield particularly in the first harvest. The plant height was higher with organic amendments including BSF frass treatment 10 t/ha and 15 t/ha compared to inorganic fertilizers and controls. This finding supports previous studies indicating that organic fertilizers improve plant growth by enhancing soil nutrients availability and microbial activity (Adesina et al., 2020). The greater plant height observed in the first harvest compared to that of the second one may be attributed to initial nutrient availability, which declined over time due to plant uptake and potential leaching losses (Xu et al., 2020). No significant difference was observed between poultry manure and inorganic fertilizers for African eggplant height in first season. This result confirmed those of Olanipon et al., 2020 who reported similar plant height on African eggplant when using poultry manure and inorganic fertilizer. The reduced plant height in the second harvest across all treatments suggests nutrient depletion over time and was consistent with the findings of Agele et al. (2018) and Vanlauwe et al. (2015), who observed that repeated harvesting without adequate nutrient replenishment led to a decline in plant vigor and productivity. Furthermore, the lowest plant height recorded under the control treatment underscores the necessity of fertilizer application for optimal African eggplant growth, as observed by Olanipon et al. (2020). Significant reductions in growth parameters were obtained when Solanum crops were grown without nutrient supplementation (Olanipon et al., 2020).

The number of leaves and leaf dimensions followed a similar trend to plant height, reinforcing the role of nutrient-rich amendments in promoting plant growth. The BSF frass (T2) consistently produced the highest leaf number and size, suggesting its high potential in supporting robust foliage development. The correlation with soil nutrient status is evident, as T2 presented the highest total N, NO_3_-, and NH_4_+ content. Nitrogen is essential for plant growth, and its higher levels in BSF frass-treated plots may explain the higher leaf and stem development observed (Sarker et al., 2020). The treatment T1 (BSF frass at 15 t/ha) and T4 (poultry manure at 10 t/ha) also promoted substantial leaf development, highlighting their efficacy in enhancing foliar growth. These findings align with previous research indicating that organic matter-rich amendments enhance photosynthetic area, thereby supporting overall plant growth (Sawinska et al., 2024; Vanlauwe et al., 2015). Chicken manure (T4) also maintained a relatively high leaf number in the second harvest, indicating its sustained nutrient release compared to mineral fertilizers, which primarily provide an immediate nutrient boost but lack long-term effects (Chen et al., 2018). This suggests that chicken manure provided a sustained effect on leaf production, consistent with the findings of Nathaniel et al. (2019), who reported that poultry manure improved leaf retention and overall canopy development in vegetable crops. The unfertilized plots gave the lowest number and size of leaves regardless of the harvest as reported by Olanipon et al. (2020).

Leaf length significantly varied between treatments, with T2 consistently producing the longest leaves (31.77 ± 0.87 cm in Harvest 1 and 19.63 ± 0.76 cm in Harvest 2). The results support studies by Ravindran et al. (2019), who found that organic fertilizers promote leaf expansion, contributing to higher biomass accumulation. The wider leaves observed in T2 (22.42 ± 0.81 cm in Harvest 1 and 11.81 ± 0.53 cm in Harvest 2) further reinforce the beneficial effects of BSF frass at 10 t/ha, as previously demonstrated by Beesigamukama et al. (2021) and Ndor et al. (2010).

Yield data further supports the positive impact of fertilizer application on African eggplant productivity. Highly significant differences in yield were observed during the first harvest, with BSF frass at 10 t/ha and 15 t/ha producing the highest yields. This aligns with the findings of Beesigamukama et al. (2021), who found that BSF frass enhances biomass accumulation due to its high nutrient content and slow-release properties. Furthermore, studies reported by Ayeni et al. (2014) and Ndor et al. (2010) confirm that the use of organic amendments contributes significantly to yield improvements in Solanum species. Lower application rates (5 t/ha) resulted in yields comparable to those obtained with chicken manure (10 t/ha) and NPK + urea, suggesting that a threshold exists for optimal nutrient supplementation, as observed by Akanbi et al. (2005) and Nathaniel et al. (2019). Similar findings were reported by Adeniyan et al. (2011), who found that organic fertilizers outperformed mineral fertilizers in promoting higher yields due to their positive effects on soil structure and microbial activity. The unfertilized plots produced the lowest yields, further supporting the necessity of fertilizer application for maintaining productivity. Interestingly, the combination of 200 kg/ha of NPK and 100 kg/ha of urea exhibited stable performance across both harvests, which aligns with previous studies indicating that synthetic fertilizers provide consistent, however they are less sustainable compared to organic amendments (Mahuku et al., 2019).

The higher performance of BSF frass treatments can be linked to increased soil nutrient availability, particularly N, P, and K, which were highest in these treatments. The results align with the findings of Beesigamukama et al. (2021), who demonstrated that black soldier fly frass significantly improved plant growth because of its high nutrient content and slow-release properties. Nurfikari (2022) observed rapid mineralization and release of plant available N due to the chitin content of the frass and easily degradable components with high N content, increasing numbers of chitin-degrading bacteria (e.g. some Gammaproteobacteria) and somewhat increasing numbers of fast-growing high N containing fungi (e.g. Mortierellomycota) were found in frass amended soils. She concludes that insect waste streams have potential as soil health-promoting amendments. Similar benefits of organic fertilizers have been reported by Mahuku et al. (2019), who highlighted that organic amendments enhance soil microbial activity and structure, leading to better nutrient uptake and overall plant health. Additionally, the positive effect of organic fertilizers compared to synthetic fertilizers corroborates the findings of Adeniyan et al. (2011), who found that Solanum crops cultivated with organic amendments exhibited superior growth and resilience against soil degradation. However, during the second harvest, no significant differences were observed among treatments, likely due to nutrient depletion over time and possible nutrient immobilization by soil microorganisms reinforcing the findings of Agele et al. (2018) and Vanlauwe et al. (2015).

Additionally, soil chemical analysis further supports the observed plant growth and yield trends. The highest total N, NO_3_-, and NH_4_+ levels in T2 indicate that BSF frass amendments contribute significantly to N enrichment. This is consistent with the findings of Gajalakshmi and Abbasi (2004), who reported that organic fertilizers, particularly insect-based frass, improve N mineralization and microbial activity. Additionally, the highest K and P levels in T3 and T2 suggest that BSF frass enhances the availability of these essential macronutrients, which play a key role in root development and reproductive growth (Roy et al., 2006). The stability of soil pH across treatments (pH < 7.5), with a slight decrease in BSF frass-treated plots, indicates that microbial decomposition processes may contribute to mild soil acidification, a common phenomenon in organic matter-rich soils (Zhao et al., 2021). This result support those from Pietri and Brookes (2009) who demonstrated that populations of bacteria and fungi are affected by both soil pH and inputs of C as substrates. These effects could possibly be manifested by the succession of different microorganisms or activities during the decomposition of the added organic matter.

Principal component analysis of S. macrocarpon parameters also showed positive correlation between EC, plant growth and yield in BSF treatments (T2, T1), compared to control and poultry manure reflecting increased ion availability and nutrient dissolution. The relatively lower EC values in control and chicken manure-treated plots (T0, T4) suggest lower nutrient solubility and availability in these treatments. This is in line with principal component analysis of African eggplant parameters and the study by Alam et al. (2020) and Mokolobate and Haynes (2002), which highlighted that organic fertilizers with higher nutrient content led to increased soil conductivity, positively correlating with improved plant performance. Naser El Deen et al. (2023) indicated that OM content in BSF frass is also higher than all other manure and compost types. N content and C/N ratio are closest to the values of cow slurry, while P content is most comparable to that of pig slurry and K content is most comparable to that of poultry manure. 

Overall, our findings highlight the agronomic benefits of organic amendments, particularly BSF frass and poultry manure, in enhancing L. sativa and S. macrocarpon growth and maintaining soil fertility. The results suggest that integrating organic amendments, notably BSF frass, into fertilization strategies can optimize nutrient availability while mitigating the environmental drawbacks associated with synthetic fertilizers. This approach directly contributes to achieving key sustainability goals in Sub-Saharan Africa (SSA), including the promotion of climate-resilient agriculture, the preservation of soil health, and the reduction of dependence on imported chemical inputs (Sachs et al., 2024). Furthermore, such practices would contribute to food security goals by improving smallholder productivity, enhancing soil fertility for sustained production, and supporting diversification through the use of locally available organic resources (Van Huis et al., 2021).

These results also reinforce regional and continental commitments, such as the Malabo Declaration and CAADP, which emphasize sustainable intensification, reduction of land degradation, and resilience building for food and nutrition security (NEPAD, 2014). Future studies should focus on long-term effects of these amendments on soil microbial dynamics and crop productivity across different agro-ecological conditions, thereby supporting broader efforts to achieve environmental sustainability and food security in Benin.

## 5 Conclusion

Residues from industrial insect production, called frass, can be an alternative to poultry manure, and inorganic fertilizers in leafy vegetable production. The optimal doses of 10 t/ha and 20 t/ha of BSF frass can be used to increase plant growth and yield in *S. macrocarpon* and *L. sativa*, respectively. The high electrical conductivity obtained in soil treated with frass confirm his contribution to optimal nutrient availability and enhancing soil health and plant growth. The lowest performance observed in soil treated with BSF Frass and inorganic fertilizers demonstrated compared to soil treated with BSF Frass alone supported the promising solutions for sustainable agriculture. Leafy greens are important staple crops, produced worldwide; Efforts to increase sustainability of these crops can improve the accessibility of fresh, nutrient dense food. Mitigating practices to improve long term environmental outcomes of controlled environment agriculture can create long term production solutions.

## Data Availability

The raw data supporting the conclusions of this article will be made available by the authors, without undue reservation.

## References

[B1] Abu E. Kwoseh C. Moses E. (2024). Peri-urban lettuce production in the Kumasi metropolis: diseases and farmers’ management strategies. Ghana J. Agric. Sci. 59, 20–30.

[B2] Adango E. Onzo A. Kassa J. W. (2021). Comportement de quelques variétés de la grande morelle, *Solanum macrocarpon* L.(Gboma) face à l’attaque de l’acarien tarsonème, *Polyphagotarsonemus latus* Banks (Acari: Tarsonemidae) au Sud-Bénin. The Journal of Animal and Plant Sciences 47 (1), 8372–8386. https://www.m.elewa.org/Journals/wp-content/uploads/2021/01/3.Adango.pdf.

[B3] Adekiya A. O. Agbede T. M. Aboyeji C. M. Dunsin O. Simeon V. T. (2019). Effects of biochar and poultry manure on soil characteristics and the yield of radish. Scientia Hortic. 243, 457–463. doi: 10.1016/j.scienta.2018.08.048

[B4] Adeniyan O. N. Ojo A. O. Akinbode O. A. Adediran J. A. (2011). Comparative study of different organic manures and NPK fertilizer for improvement of soil chemical properties and dry mater yield of maize in two different soils. J. Soil Sci. Environ. Manage. 2, 9–13.

[B5] Adesina I. Bhowmik A. Sharma H. Shahbazi A. (2020). A review on the current state of knowledge of growing conditions, agronomic soil health practices and utilities of hemp in the United States. Agriculture 10, 129. doi: 10.3390/agriculture10040129

[B6] Adewale O. B. Oloyede O. I. Onasanya A. Olayide I. I. Anadozie S. O. Fadaka A. O. (2015). Hepatoprotective effect of aqueous extract of Solanum macrocarpon leaves against carbon tetrachloride-induced liver damage in rats. J. Appl. Pharm. Sci. 5, 81–86. doi: 10.7324/JAPS.2015.58.S13

[B7] Agele S. Aiyelari P. Famuwagun B. Adegboye J. Oyeneyin E. (2018). Effects of watering regime and mycorrhizal inoculation on seedling growth and drought tolerant traits of cocoa (Theobroma cacao L.) varieties. Global Journal of Botanical Science, 6 (1), 26–41.

[B8] Ahouangninou C. (2013). Durabilité de la production maraîchère au sud-Bénin : un essai de l’approche écosystémique. UAC. Thèse de doctorat, Bénin. Available online at : https://doi.org/10/1/document_572410.pdf. Retrieved on 12 mars 2025

[B9] Akanbi W. B. Togun A. O. Adediran J. A. Ilupeju E. A. O. (2005). Growth, dry matter and fruit yields components of okra under organic and inorganic sources of nutrients. American-Eurasian J. Sustain. Agric. 2, 1–5.

[B10] Alam M. Z. Rahman M. M. Islam M. S. (2020). Principal component analysis of morphological traits in eggplant (Solanum melongena L.) genotypes. Asian J. Res. Crop Sci. 5, 1–9.

[B11] Alromian F. M. (2020). Effect of type of compost and application rateon growth and quality of lettuce plant. J. Plant Nutr. 43, 2797–2809. doi: 10.1080/01904167.2020.1795034 doi: 10.17170/kobra-202210116965

[B12] Assogba C. G. Vodouhê G. T. Adje B. Dassou A. Tovignan S. D. Kindomihou V. . (2022). Agroecological transition in vegetable farming systems in southern Benin. Lessons from a diagnostic analysis. J. Agric. Rural Dev. Tropics Subtropics (JARTS) 123, 205–214.

[B13] Ayeni L. S. (2014). Effect of Manufactured Organic Fertilizers on Soil Chemical Properties and Yield of Tomato (Lycopersicum lycopersicon) in Alfisol, Southwestern Nigeria. Molecular Soil Biology 5 (6), 1–6. doi: 10.5376/msb.2014.05.0006

[B14] Aytenew M. Bore G. (2020). Effects of organic amendments on soil fertility and environmental quality: A review. Plant Sci. 8, 112–119. doi: 10.11648/j.jps.20200805.12

[B15] Banks I. J. Gibson W. T. Cameron M. M. (2014). Growth rates of black soldier fly larvae fed on fresh human faeces and their implication for improving sanitation. Trop. Med. Int. Health 19, 14–22. doi: 10.1111/tmi.12228, PMID: 24261901

[B16] Beesigamukama D. Mochoge B. Korir N. K. K. M. Fiaboe K. Nakimbugwe D. Khamis F. M. . (2021). Low-cost technology for recycling agro-industrial waste into nutrient-rich organic fertilizer using black soldier fly. Waste Manage. 119, 183–194. doi: 10.1016/j.wasman.2020.09.043, PMID: 33068885

[B17] Beesigamukama D. Mochoge B. Korir N. K. Fiaboe K. K. Nakimbugwe D. Khamis F. M. . (2020). Exploring black soldier fly frass as novel fertilizer for improved growth, yield, and nitrogen use efficiency of maize under field conditions. Front. Plant Sci. 11, 574592. doi: 10.3389/fpls.2020.574592, PMID: 33072150 PMC7539147

[B18] Chavez M. Y. Uchanski M. Tomberlin J. K. (2023). Impacts des excréments larvaires de la mouche soldat noire (Diptera: Stratiomyidae) sur la production de tomates. J. Economic Entomol. 116, 1490–1495. doi: 10.1093/jee/toad150, PMID: 37494678

[B19] Chavez M. Y. Uchanski M. Tomberlin J. K. (2024). Impacts of black soldier fly, *Hermetia illucens*, larval frass on lettuce and arugula production. Front. Sustain. Food Syst. 8. doi: 10.3389/fsufs.2024.1399932

[B20] Chen Y. Camps-Arbestain M. Shen Q. Singh B. Cayuela M. L. (2018). The long-term role of organic amendments in building soil nutrient fertility: a meta-analysis and review. Nutrient Cycling Agroecosystems 111, 103–125. doi: 10.1007/s10705-017-9903-5

[B21] de Bruin S. Dengerink J. van Vliet J. (2021). Urbanisation as driver of food system transformation and opportunities for rural livelihoods. Food Secur. 13, 781–798. doi: 10.1007/s12571-021-01182-8, PMID: 34221191 PMC8237550

[B22] De Vos K. Janssens C. Jacobs L. Campforts B. Boere E. Kozicka M. . (2024). African food system and biodiversity mainly affected by urbanization via dietary shifts. Nat. Sustainability 7, 869–878. doi: 10.1038/s41893-024-01362-2

[B23] Dougnon T. V. Bankolé H. S. Johnson R. C. Klotoé J. R. Dougnon G. Gbaguidi F. . (2012). Phytochemical screening, nutritional and toxicological analyses of leaves and fruits of *solanum macrocarpon* linn (Solanaceae) in cotonou (Benin). Food and Nutrition Sciences 3 (11), 1595–1603. doi: 10.4236/fns.2012.311208

[B24] Dzepe D. Nana P. Fotso A. Tchuinkam T. Djouaka R. (2020). Influence of larval density, substrate moisture content and feedstock ratio on life history traits of black soldier fly larvae. J. Insects as Food Feed 6, 133–140. doi: 10.3920/JIFF2019.0034

[B25] Eggink K. M. Lund I. Pedersen P. B. Hansen B. W. Dalsgaard J. (2022). Biowaste and by-products as rearing substrates for black soldier fly (*Hermetia illucens*) larvae: Effects on larval body composition and performance. PloS One 17, e0275213. doi: 10.1371/journal.pone.0275213, PMID: 36174084 PMC9521838

[B26] Elissen H. van der Weide R. Gollenbeek L. (2023). Effects of black soldier fly frass on plant and soil characteristics: a literature overview. Wageningen Research, Wageningen. doi: 10.18174/587213

[B27] FAO (2017). The future of food and agriculture – Trends and challenges. Food and Agriculture Organization of the United Nations, Rome.

[B28] Fernández-Romero M. L. Clark J. M. Collins C. D. Parras-Alcántara L. Lozano-García B. (2016). Evaluation of optical techniques for characterising soil organic matter quality in agricultural soils. Soil Tillage Res. 155, 450–460. doi: 10.1016/j.still.2015.05.004

[B29] Gajalakshmi S. Abbasi S. A. (2004). Neem leaves as a source of fertilizer-cum-pesticide vermicompost. Bioresource Technol. 92, 291–296. doi: 10.1016/j.biortech.2003.09.012, PMID: 14766163

[B30] Gärttling D. Kirchner S. M. Schulz H. (2020). Assessment of the N-and P-fertilization effect of black soldier fly (Diptera: Stratiomyidae) by-products on maize. J. Insect Sci. 20, 8. doi: 10.1093/jisesa/ieaa089, PMID: 32960967 PMC7508297

[B31] Gbaguidi A. A. Assogba P. Dansi M. Yedomonhan H. Dansi A. (2015). Caractérisation agromorphologique des variétés de niébé cultivées au Bénin. Int. J. Biol. Chem. Sci. 9, 1050–1066. doi: 10.4314/ijbcs.v9i2.40

[B32] Habwe F. O. Wanlingo K. M. Onyango M. O. A. (2008). Food processing and preparation technologies for sustainable utilization of African indigenous vegetables for nutrition security and wealth creation in Kenya. In: Robertson G. L. Lupien J. R. (Editors), Using food science and technology to improve nutrition and promote national development, Ch 13. International Union of Food Science and Technology, Ontario, Canada.

[B33] Houben D. Daoulas G. Faucon M. P. Dulaurent A. M. (2020). Potential use of mealworm frass as a fertilizer: Impact on crop growth and soil properties. Sci. Rep. 10, 4659. doi: 10.1038/s41598-020-61765-x, PMID: 32170150 PMC7069999

[B34] Houessou M. D. Cassee A. Sonneveld B. G. (2021). The effects of the COVID-19 pandemic on food security in rural and urban settlements in Benin: do allotment gardens soften the blow? Sustainability 13, 7313. doi: 10.3390/su13137313

[B35] Houessou M. D. van de Louw M. Sonneveld B. G. (2020). What constraints the expansion of urban agriculture in Benin? Sustainability 12, 5774. doi: 10.3390/su12145774

[B36] Ibiam O. F. Nwigwe I. (2013). The effect of fungi associated with leaf blight of Solanum aethiopicum L. in the field on the nutrient and phytochemical composition of the leaves and fruits of the plant. J. Plant Pathol. Microbiol. 4, 191–195. doi: 10.4172/2157-7471.1000191

[B37] Idris M. Rismayani D. Aulia A. Nopiyanti T. Rahayu R. (2024). Biology of Black Soldier Fly (Hermetia illucens) and Utilization of its Waste (Maggot Frass) for Plant Growth: A Literature Review. Jurnal Biologi Tropis 24, 273–291. doi: 10.29303/jbt.v24i3.7226

[B38] Institut national de la statistique et de l’analyse économique (2016). Cahier des villages et quartiers de ville du département de […]. Recensement général de la population et de l’habitation (RGPH-4, 2013). INSAE.

[B39] Kenny O. O'Beirne D. (2009). The effects of washing treatment on antioxidant retention in ready-to-use iceberg lettuce. International Journal of Food Science and Technology 44, 1146–1156.

[B40] Khan M. A. Adnan M. Basir A. Fahad S. Hafeez A. Saleem M. H. . (2023). Impact of tillage and potassium levels and sources on growth, yield and yield attributes of wheat. Pakistan J. Bot. 55, 321–326. doi: 10.30848/PJB2023-1(30)

[B41] Kihara A. (2016). Synthesis and degradation pathways, functions, and pathology of ceramides and epidermal acylceramides. Prog. Lipid Res. 63, 50–69. doi: 10.1016/j.plipres.2016.04.001, PMID: 27107674

[B42] Kim M. J. Moon Y. Tou J. C. Mou B. Waterland N. L. (2016). Nutritional value, bioactive compounds and health benefits of lettuce (Lactuca sativa L.). Journal of Food Composition and Analysis 49, 19–34.

[B43] Klammsteiner T. Turan V. Fernández-Delgado Juárez M. Insam H. (2020). Suitability of black soldier fly frass as soil amendment and implications for organic waste hygienization. Agronomy 10, 1578. doi: 10.3390/agronomy10101578

[B44] Lalander C. Diener S. Magri M. E. Zurbrügg C. Lindström A. Vinnerås B. (2013). Faecal sludge management with the larvae of the black soldier fly (Hermetia illucens)—From a hygiene aspect. Sci. Total Environ. 458, 312–318. doi: 10.1016/j.scitotenv.2013.04.033, PMID: 23669577

[B45] Lawrence A. Nehler T. Andersson E. Karlsson M. Thollander P. (2019). Drivers, barriers and success factors for energy management in the Swedish pulp and paper industry. J. cleaner production 223, 67–82. doi: 10.1016/j.jclepro.2019.03.143

[B46] Liu Y. Lan X. Hou H. Ji J. Liu X. Lv Z. (2024). Multifaceted ability of organic fertilizers to improve crop productivity and abiotic stress tolerance: review and perspectives. Agronomy 14, 1141. doi: 10.3390/agronomy14061141

[B47] Liverpool-Tasie L. S. O. Omonona B. T. Sanou A. Ogunleye W. O. (2017). Is increasing inorganic fertilizer use for maize production in SSA a profitable proposition? Evidence from Nigeria. Food Policy 67, 41–51. doi: 10.1016/j.foodpol.2016.09.011, PMID: 28413245 PMC5384440

[B48] LoMonaco G. Franco A. De Smet J. Scieuzo C. Salvia R. Falabella P. (2024). Larval frass of Hermetia illucens as organic fertilizer: composition and beneficial effects on different crops. Insects 15, 293. doi: 10.3390/insects15040293, PMID: 38667423 PMC11050032

[B49] Lopes I. G. Yong J. W. Lalander C. (2022). Frass derived from black soldier fly larvae treatment of biodegradable wastes. A critical review and future perspectives. Waste Manage. 142, 65–76. doi: 10.1016/j.wasman.2022.02.007, PMID: 35176600

[B50] Ludemann C. I. Wanner N. Chivenge P. Dobermann A. Einarsson R. Grassini P. . (2024). A global FAOSTAT reference database of cropland nutrient budgets and nutrient use efficiency, (1961–2020): nitrogen, phosphorus and potassium. Earth Syst. Sci. Data 16, 525–541. doi: 10.5194/essd-16-525-2024

[B51] Mahuku G. Nzioki H. S. Mutegi C. Kanampiu F. Narrod C. Makumbi D. (2019). Pre-harvest management is a critical practice for minimizing aflatoxin contamination of maize. Food Control 96, 219–226. doi: 10.1016/j.foodcont.2018.08.032, PMID: 30713368 PMC6251936

[B52] Mensah A. K. Frimpong K. A. (2018). Biochar and/or compost applications improve soil properties, growth, and yield of maize grown in acidic rainforest and coastal savannah soils in Ghana. Int. J. Agron. 2018, 6837404. doi: 10.1155/2018/6837404

[B53] Mokolobate M. Haynes R. (2002). Comparative liming effect of four organic residues applied to an acid soil. Biol. fertility soils 35, 79–85. doi: 10.1007/s00374-001-0439-z

[B54] Muhanji G. Roothaert R. L. Webo C. Stanley M. (2011). African indigenous vegetable enterprises and market access for small-scale farmers in East Africa. Int. J. Agric. Sustainability 9, 194–202. doi: 10.3763/ijas.2010.0561

[B55] Nacoulma J. D. Guigma J. B. (2015). Institutional context of soil information in Benin. Available online at: https://cgspace.cgiar.org/server/api/core/bitstreams/9ecfe8ec-56b3-4a8d-960b-5fc6536cc1f5/content (Accessed December 15, 2025).

[B56] Nájera Espinosa S. Hadida G. Jelmar Sietsma A. Alae-Carew C. Turner G. Green R. . (2024). Mapping the evidence of novel plant-based foods: a systematic review of nutritional, health, and environmental impacts in high-income countries. Nutr. Rev. 83 (7), e1626–e1646. doi: 10.5281/zenodo.7116157, PMID: 38657969 PMC12166169

[B57] Naser El Deen S. van Rozen K. Elissen H. van Wikselaar P. Fodor I. van der Weide R. . (2023). Bioconversion of different waste streams of animal and vegetal origin and manure by black soldier fly larvae Hermetia illucens L. (Diptera: Stratiomyidae). Insects 14, 204. doi: 10.3390/insects14020204, PMID: 36835773 PMC9968099

[B58] Nathaniel S. Nwodo O. Adediran A. Sharma G. Shah M. Adeleye N. (2019). Ecological footprint, urbanization, and energy consumption in South Africa: including the excluded. Environ. Sci. pollut. Res. 26, 27168–27179. doi: 10.1007/s11356-019-05924-2, PMID: 31321720

[B59] Ndambi O. A. Pelster D. E. Owino J. O. De Buisonjé F. Vellinga T. (2019). Manure management practices and policies in sub-Saharan Africa: implications on manure quality as a fertilizer. Front. Sustain. Food Syst. 3, 29. doi: 10.3389/fsufs.2019.00029

[B60] Ndor E. Agbede O. O. Dauda S. N. (2010). Growth and Yield Response of cotton (*Gossypium* spp) to varying levels of nitrogen and phosphorus fertilization in southern Guinea savanna zone, Nigeria. PAT 6, 119–125. doi: 10.3390/insects14020204, PMID: 36835773 PMC9968099

[B61] NEPAD (2014). Malabo Declaration on Accelerated Agricultural Growth and Transformation for Shared Prosperity and Improved Livelihoods, NEPAD Agency, Midrand (South Africa).

[B62] Nurfikari A. (2022). Prospects of residual streams from insect cultivation for sustainable crop management. Doctoral dissertation, Wageningen University, Wageningen.

[B63] Nwezoku U. Milala M. A. Gidado A. A. (2017). Antioxidant and hepatoprotective effect of extracts of some commonly consumed local fruits in Maiduguri. Journal of Pharmaceutical and Biomedical Sciences 7 (4), 85–89.

[B64] Olanipon D. G. Kayode J. Ayeni M. J. (2020). Growth, yield, nutritional and mineral composition of Solanum macrocarpon L. as affected by fertilizer application. J. Biotechnol. Res. 6, 69–78. doi: 10.32861/jbr.66.69.78

[B65] Ossamulu I. F. Akanya H. O. Jigam A. A. Egwim E. C. (2014). Nutrient and phytochemical constituents of four eggplant varieties. Food Sci. 73, 26424–26428.

[B66] Osuagwu G. G. E. Edeoga H. O. (2010). Effect of fertilizer treatment on the antimicrobial activity of the leaves of Ocimum gratissimum (L.) and Gongronema latifolium (Benth). Afr. J. Biotechnol. 9, 8918–8922.

[B67] Palma L. Fernández-Bayo J. Putri F. VanderGheynst J. S. (2020). Almond by-product composition impacts the rearing of black soldier fly larvae and quality of the spent substrate as a soil amendment. J. Sci. Food Agric. 100, 4618–4626. doi: 10.1002/jsfa.10522, PMID: 32419145 PMC7496255

[B68] Panwar S. S. Chauhan J. K. Noopur K. Panwar N. Pradhan K. Kumar L. . (2024). Disease prevention in human through bioactive medicinal molecules of vegetables: A review. Indian Res. J. Ext. Edu 24, 95–102. doi: 10.54986/irjee/2024/apr-jun/95-105

[B69] Pietri J. A. Brookes P. C. (2009). Substrate inputs and pH as factors controlling microbial biomass, activity and community structure in an arable soil. Soil Biol. Biochem. 41, 1396–1405. doi: 10.1016/j.soilbio.2009.03.017

[B70] Poveda J. González-Andrés F. (2021). Bacillus as a source of phytohormones for use in agriculture. Appl. Microbiol. Biotechnol. 105 (23), 8629–8645., PMID: 34698898 10.1007/s00253-021-11492-8

[B71] Ravindran B. Lee S. R. Chang S. W. Nguyen D. D. Chung W. J. Balasubramanian B. . (2019). Positive effects of compost and vermicompost produced from tannery waste-animal fleshing on the growth and yield of commercial crop-tomato (Lycopersicon esculentum L.) plant. Journal of Environmental Management 234, 154–158. doi: 10.1016/j.jenvman.2018.12.100, PMID: 30616187

[B72] Rakotoson T. Johnson J. M. Senthilkumar K. Ibrahim A. Saito K. (2025). On-farm nitrogen, phosphorus, and potassium partial balances in three major rice production systems in sub-Saharan Africa. Field Crops Res. 322, 109714. doi: 10.1016/j.fcr.2024.109714

[B73] Roy R. N. Finck A. Blair G. J. Tandon H. L. S. (2006). *Plant nutrition for food security: A guide for integrated nutrient management* (FAO Fertilizer and Plant Nutrition Bulletin No. 16). Food and Agriculture Organization of the United Nations, Rome.

[B74] Rusinamhodzi L. Corbeels M. Giller K. E. (2016). Diversity in crop residue management across an intensification gradient in southern Africa: System dynamics and crop productivity. Field Crops Res. 185, 79–88. doi: 10.1016/j.fcr.2015.10.007

[B75] Sachs J. D. Lafortune G. Fuller G. (2024). The SDGs and the UN summit of the future. Sustainable Development Report 2024. Paris: SDSN, Dublin University Press, Dublin. doi: 10.25546/108572

[B76] Salehi F. (2021). Quality, physicochemical, and textural properties of dairy products containing fruits and vegetables: A review. Food Sci. Nutr. 9, 4666–4686. doi: 10.1002/fsn3.2430, PMID: 34401112 PMC8358338

[B77] Santos C. Agbangla C. Chougourou D. Missihoun A. A. Ahanhanzo C. (2015). Influence of agro ecology on rice varietal resistance to Sitophilus oryzae (Coleoptera: Curculionidae) and Sitotroga cerealella (Lepidoptera: Gelechiidae) in Benin. Am. J. Plant Sci. 6, p.2832. doi: 10.4236/ajps.2015.618280

[B78] Sarker A. Deepo D. M. Nandi R. Rana J. Islam S. Rahman S. . (2020). A review of microplastics pollution in the soil and terrestrial ecosystems: A global and Bangladesh perspective. Science of the Total Environment 733, 139296. doi: 10.1016/j.scitotenv.2020.139296, PMID: 32473463

[B79] SAS Institute Inc (2009). SAS^®^ 9.2 Macro Language: Reference (Cary, NC: SAS Institute Inc).

[B80] Sawinska Z. Radzikowska-Kujawska D. Kowalczewski P.Ł. Grzanka M. Sobiech Ł. Skrzypczak G. . (2024). Hermetia illucens Frass Fertilization: A Novel Approach for Enhancing Lettuce Resilience and Photosynthetic Efficiency under Drought Stress Conditions. Appl. Sci. 14, 2386. doi: 10.3390/app14062386

[B81] Soetan K. O. Olaiya C. O. Oyewole O. E. (2010). The Importance of Mineral Elements for Humans, Domestic Animals and Plants: A Review. African Journal of Food Science 4, 200–222.

[B82] Sun S.-S. Liu X.-P. Zhao X.-Y. Medina-Roldán E. He Y.-H. Lv P. . (2022). Annual herbaceous plants exhibit altered morphological traits in response to altered precipitation and drought patterns in semiarid sandy grassland, northern China. Front. Plant Sci. 13. doi: 10.3389/fpls.2022.756950, PMID: 35812936 PMC9260268

[B83] Surendra K. C. Olivier R. Tomberlin J. K. Jha R. Khanal S. K. (2016). Bioconversion of organic wastes into biodiesel and animal feed via insect farming. Renewable Energy 98, 197–202. doi: 10.1016/j.renene.2016.03.022

[B84] van Huis A. Rumpold B. A. van der Fels-Klerx H. J. Tomberlin J. K. (2021). Advancing edible insects as food and feed in a circular economy. J. Insects as Food Feed 7, 935–948. doi: 10.3920/JIFF2021.x005

[B85] Vanlauwe B. Descheemaeker K. Giller K. E. Huising J. Merckx R. Nziguheba G. . (2015). Integrated soil fertility management in sub-Saharan Africa: Unravelling local adaptation. SOIL 1, 491–508. doi: 10.5194/soil-1-491-2015

[B86] Watson C. Houben D. Wichern F. (2022). Editorial: Frass: The Legacy of Larvae Benefits and Risks of Residues From Insect Production. Frontiers in Sustainable Food Systems 6, 889004. doi: 10.3389/fsufs.2022.889004

[B87] Watson C. Schlösser C. Vögerl J. Wichern F. (2021). Excellent excrement? Frass impacts on a soil’s microbial community, processes and metal bioavailability. Appl. Soil Ecol. 168, 104110. doi: 10.1016/j.apsoil.2021.104110

[B88] Wuenscher R. Unterfrauner H. Peticzka R. Zehetner F. (2015). A comparison of 14 soil phosphorus extraction methods applied to 50 agricultural soils from Central Europe. Plant Soil Environment, 61 (2), 8696. doi: 10.17221/932/2014-PSE

[B89] Xu J. Cai H. Wang X. Ma C. Lu Y. Ding Y. . (2020). Exploring optimal irrigation and nitrogen fertilization in a winter wheat-summer maize rotation system for improving crop yield and reducing water and nitrogen leaching. Agric. Water Manage. 228, 105904. doi: 10.1016/j.agwat.2019.105904

[B90] Zhao Q. Guo Y. Ye T. Gasparrini A. Tong S. Overcenco A. . (2021). Global, regional, and national burden of mortality associated with non-optimal ambient temperatures from 2000 to 2019: A three-stage modelling study. Lancet Planetary Health 5, e415–e425. doi: 10.1016/S2542-5196(21)00134-3, PMID: 34245712

