# Correction: Black soldier fly frass as a sustainable organic fertilizer: enhancing productivity of leafy vegetables and soil health in Benin

**DOI:** 10.3389/fpls.2026.1796257

**Published:** 2026-02-09

**Authors:** Frontiers Production Office

**Affiliations:** Frontiers Media SA, Lausanne, Switzerland

**Keywords:** black soldier fly, frass, insect, yield, manure, organic

The details of the Editor were erroneously displayed as “Chrysantus Mbi Tanga, International Centre of Insect Physiology and Ecology (ICIPE), Kenya”. The correct details are “Fleurdeliz Maglangit, University of the Philippines Cebu, Philippines.”

The original version of this article has been updated.

